# Supplementary material for: Obesity-associated changes in molecular biology of primary breast cancer
Source: Nat Commun. 2023 Jul 21;14:4418. doi: 10.1038/s41467-023-39996-z (PMC10361985; doi:10.1038/s41467-023-39996-z)
Supplement: Supplementary file 1 — Supplementary Information File [file 41467_2023_39996_MOESM1_ESM.pdf]

Supplementary Figures

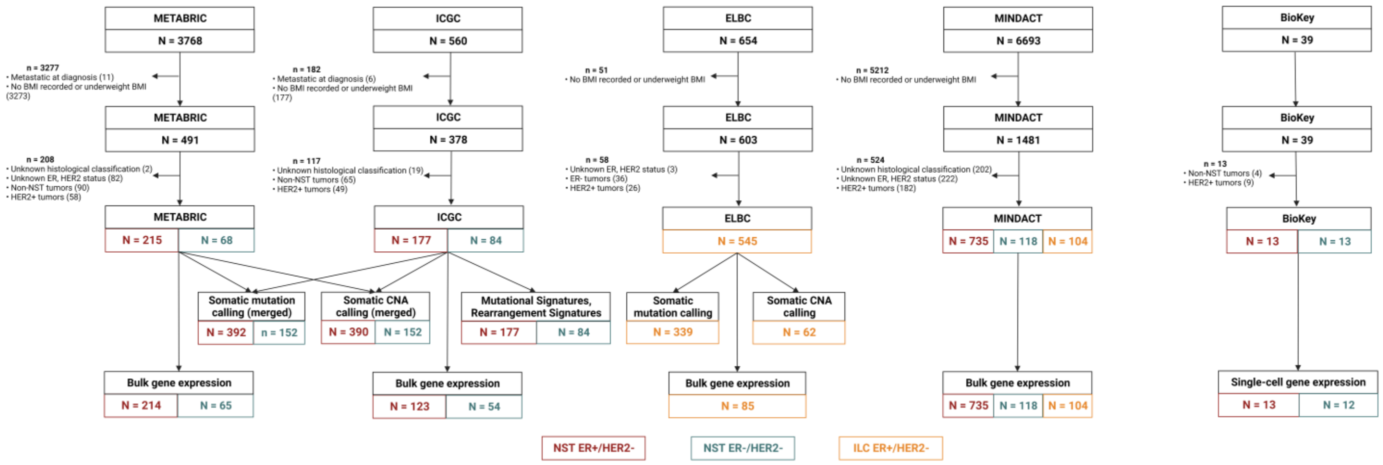

Supplementary Figure 1. Overview of the data flow in the study. Flowchart displaying the data selection and stratification process and showing the final numbers of cases analyzed in the study.

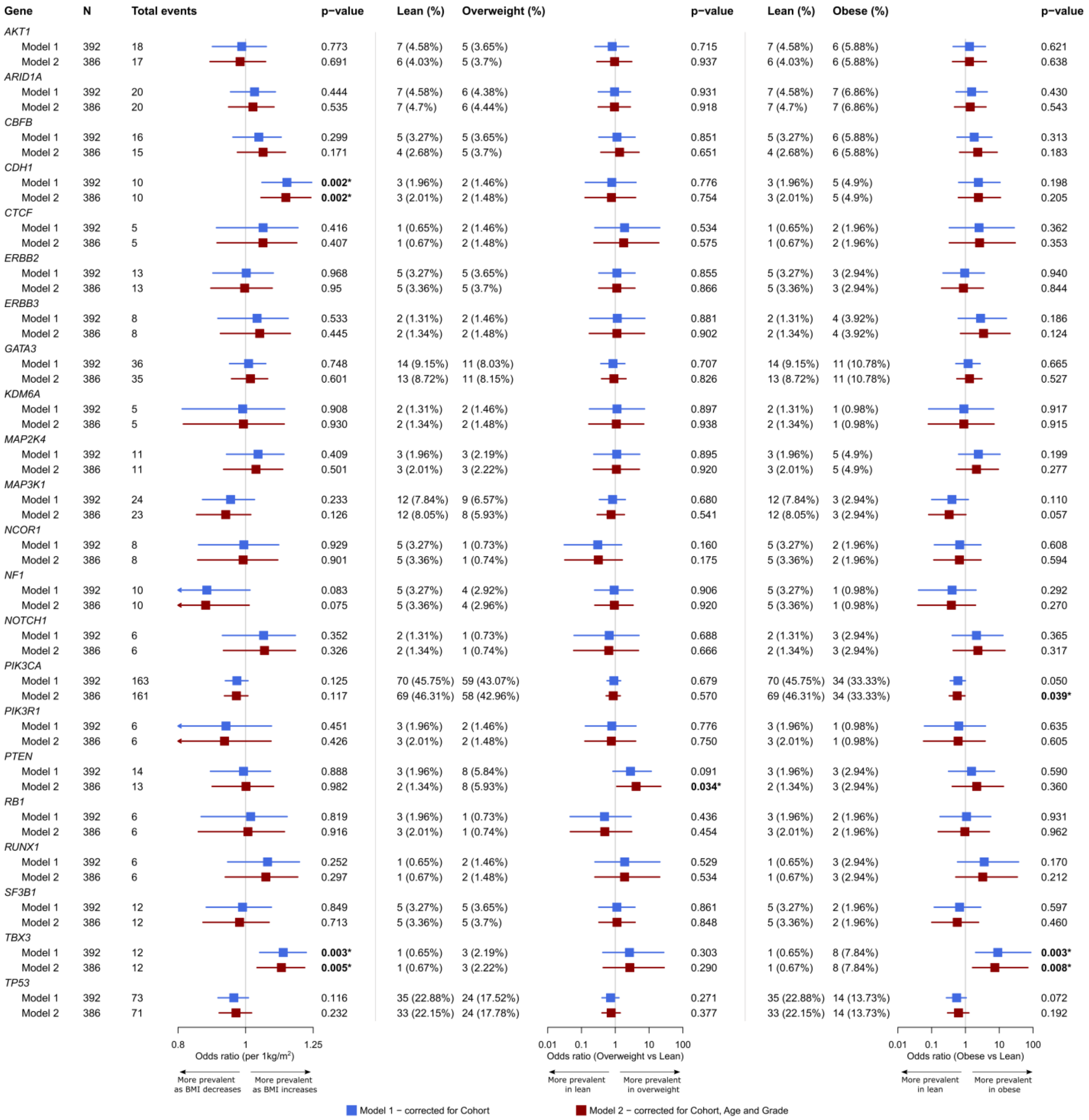

Gene	N	Total events	p–value	Lean (%)	Overweight (%)	p–value	Lean (%)	Obese (%)	p–value
AKT1									
Model 1	392	18	0.773	7 (4.58%)	5 (3.65%)	0.715	7 (4.58%)	6 (5.88%)	0.621
Model 2	386	17	0.691	6 (4.03%)	5 (3.7%)	0.937	6 (4.03%)	6 (5.88%)	0.638
ARID1A									
Model 1	392	20	0.444	7 (4.58%)	6 (4.38%)	0.931	7 (4.58%)	7 (6.86%)	0.430
Model 2	386	20	0.535	7 (4.7%)	6 (4.44%)	0.918	7 (4.7%)	7 (6.86%)	0.543
CBFB									
Model 1	392	16	0.299	5 (3.27%)	5 (3.65%)	0.851	5 (3.27%)	6 (5.88%)	0.313
Model 2	386	15	0.171	4 (2.68%)	5 (3.7%)	0.651	4 (2.68%)	6 (5.88%)	0.183
CDH1									
Model 1	392	10	**0.002***	3 (1.96%)	2 (1.46%)	0.776	3 (1.96%)	5 (4.9%)	0.198
Model 2	386	10	**0.002***	3 (2.01%)	2 (1.48%)	0.754	3 (2.01%)	5 (4.9%)	0.205
CTCF									
Model 1	392	5	0.416	1 (0.65%)	2 (1.46%)	0.534	1 (0.65%)	2 (1.96%)	0.362
Model 2	386	5	0.407	1 (0.67%)	2 (1.48%)	0.575	1 (0.67%)	2 (1.96%)	0.353
ERBB2									
Model 1	392	13	0.968	5 (3.27%)	5 (3.65%)	0.855	5 (3.27%)	3 (2.94%)	0.940
Model 2	386	13	0.95	5 (3.36%)	5 (3.7%)	0.866	5 (3.36%)	3 (2.94%)	0.844
ERBB3									
Model 1	392	8	0.533	2 (1.31%)	2 (1.46%)	0.881	2 (1.31%)	4 (3.92%)	0.186
Model 2	386	8	0.445	2 (1.34%)	2 (1.48%)	0.902	2 (1.34%)	4 (3.92%)	0.124
GATA3									
Model 1	392	36	0.748	14 (9.15%)	11 (8.03%)	0.707	14 (9.15%)	11 (10.78%)	0.665
Model 2	386	35	0.601	13 (8.72%)	11 (8.15%)	0.826	13 (8.72%)	11 (10.78%)	0.527
KDM6A									
Model 1	392	5	0.908	2 (1.31%)	2 (1.46%)	0.897	2 (1.31%)	1 (0.98%)	0.917
Model 2	386	5	0.930	2 (1.34%)	2 (1.48%)	0.938	2 (1.34%)	1 (0.98%)	0.915
MAP2K4									
Model 1	392	11	0.409	3 (1.96%)	3 (2.19%)	0.895	3 (1.96%)	5 (4.9%)	0.199
Model 2	386	11	0.501	3 (2.01%)	3 (2.22%)	0.920	3 (2.01%)	5 (4.9%)	0.277
MAP3K1									
Model 1	392	24	0.233	12 (7.84%)	9 (6.57%)	0.680	12 (7.84%)	3 (2.94%)	0.110
Model 2	386	23	0.126	12 (8.05%)	8 (5.93%)	0.541	12 (8.05%)	3 (2.94%)	0.057
NCOR1									
Model 1	392	8	0.929	5 (3.27%)	1 (0.73%)	0.160	5 (3.27%)	2 (1.96%)	0.608
Model 2	386	8	0.901	5 (3.36%)	1 (0.74%)	0.175	5 (3.36%)	2 (1.96%)	0.594
NF1									
Model 1	392	10	0.083	5 (3.27%)	4 (2.92%)	0.906	5 (3.27%)	1 (0.98%)	0.292
Model 2	386	10	0.075	5 (3.36%)	4 (2.96%)	0.920	5 (3.36%)	1 (0.98%)	0.270
NOTCH1									
Model 1	392	6	0.352	2 (1.31%)	1 (0.73%)	0.688	2 (1.31%)	3 (2.94%)	0.365
Model 2	386	6	0.326	2 (1.34%)	1 (0.74%)	0.666	2 (1.34%)	3 (2.94%)	0.317
PIK3CA									
Model 1	392	163	0.125	70 (45.75%)	59 (43.07%)	0.679	70 (45.75%)	34 (33.33%)	0.050
Model 2	386	161	0.117	69 (46.31%)	58 (42.96%)	0.570	69 (46.31%)	34 (33.33%)	**0.039***
PIK3R1									
Model 1	392	6	0.451	3 (1.96%)	2 (1.46%)	0.776	3 (1.96%)	1 (0.98%)	0.635
Model 2	386	6	0.426	3 (2.01%)	2 (1.48%)	0.750	3 (2.01%)	1 (0.98%)	0.605
PTEN									
Model 1	392	14	0.888	3 (1.96%)	8 (5.84%)	0.091	3 (1.96%)	3 (2.94%)	0.590
Model 2	386	13	0.982	2 (1.34%)	8 (5.93%)	**0.034***	2 (1.34%)	3 (2.94%)	0.360
RB1									
Model 1	392	6	0.819	3 (1.96%)	1 (0.73%)	0.436	3 (1.96%)	2 (1.96%)	0.931
Model 2	386	6	0.916	3 (2.01%)	1 (0.74%)	0.454	3 (2.01%)	2 (1.96%)	0.962
RUNX1									
Model 1	392	6	0.252	1 (0.65%)	2 (1.46%)	0.529	1 (0.65%)	3 (2.94%)	0.170
Model 2	386	6	0.297	1 (0.67%)	2 (1.48%)	0.534	1 (0.67%)	3 (2.94%)	0.212
SF3B1									
Model 1	392	12	0.849	5 (3.27%)	5 (3.65%)	0.861	5 (3.27%)	2 (1.96%)	0.597
Model 2	386	12	0.713	5 (3.36%)	5 (3.7%)	0.848	5 (3.36%)	2 (1.96%)	0.460
TBX3									
Model 1	392	12	**0.003***	1 (0.65%)	3 (2.19%)	0.303	1 (0.65%)	8 (7.84%)	**0.003***
Model 2	386	12	**0.005***	1 (0.67%)	3 (2.22%)	0.290	1 (0.67%)	8 (7.84%)	**0.008***
TP53									
Model 1	392	73	0.116	35 (22.88%)	24 (17.52%)	0.271	35 (22.88%)	14 (13.73%)	0.072
Model 2	386	71	0.232	33 (22.15%)	24 (17.78%)	0.377	33 (22.15%)	14 (13.73%)	0.192

Supplementary Figure 2. Association of BMI as a categorical variable with oncogenic mutations of breast cancer-specific driver genes in NST ER+/HER2- patients. Forest plots showing the associations evaluated using Firth's logistic regression between BMI, as a categorical variable (overweight vs lean, and obese vs lean), and driver gene mutations. Merged data from two cohorts, METABRIC and ICGC, was used. Gene mutations with at least 5 events detected in the respective cohorts were evaluated. Color-coded boxes indicate point estimates of odds ratios, and whiskers indicate their corresponding 95% confidence intervals. All statistical tests were two-sided. p-values shown were not corrected for multiple testing.

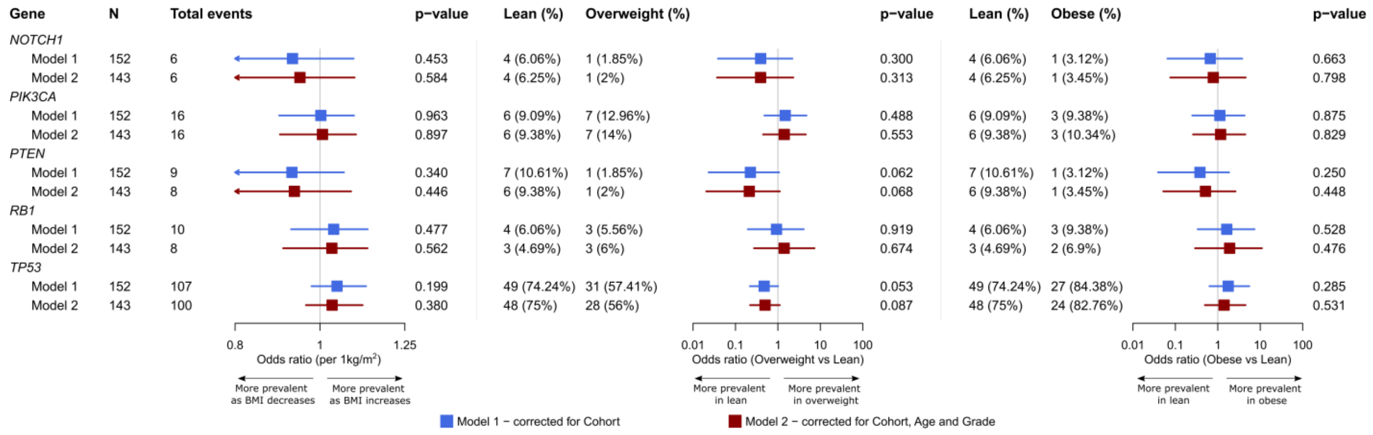

Supplementary Figure 3. Association of BMI with oncogenic mutations of breast cancer-specific driver genes in NST ER-/HER2- patients. Forest plots showing the associations evaluated using Firth's logistic regression between BMI, as a categorical variable (overweight vs lean, and obese vs lean), and driver gene mutations. Merged data from two cohorts, METABRIC and ICGC, was used. Gene mutations with at least 5 events detected in the respective cohorts were evaluated. Color-coded boxes indicate point estimates of odds ratios, and whiskers indicate their corresponding 95% confidence intervals. All statistical tests were two-sided. p-values shown were not corrected for multiple testing.

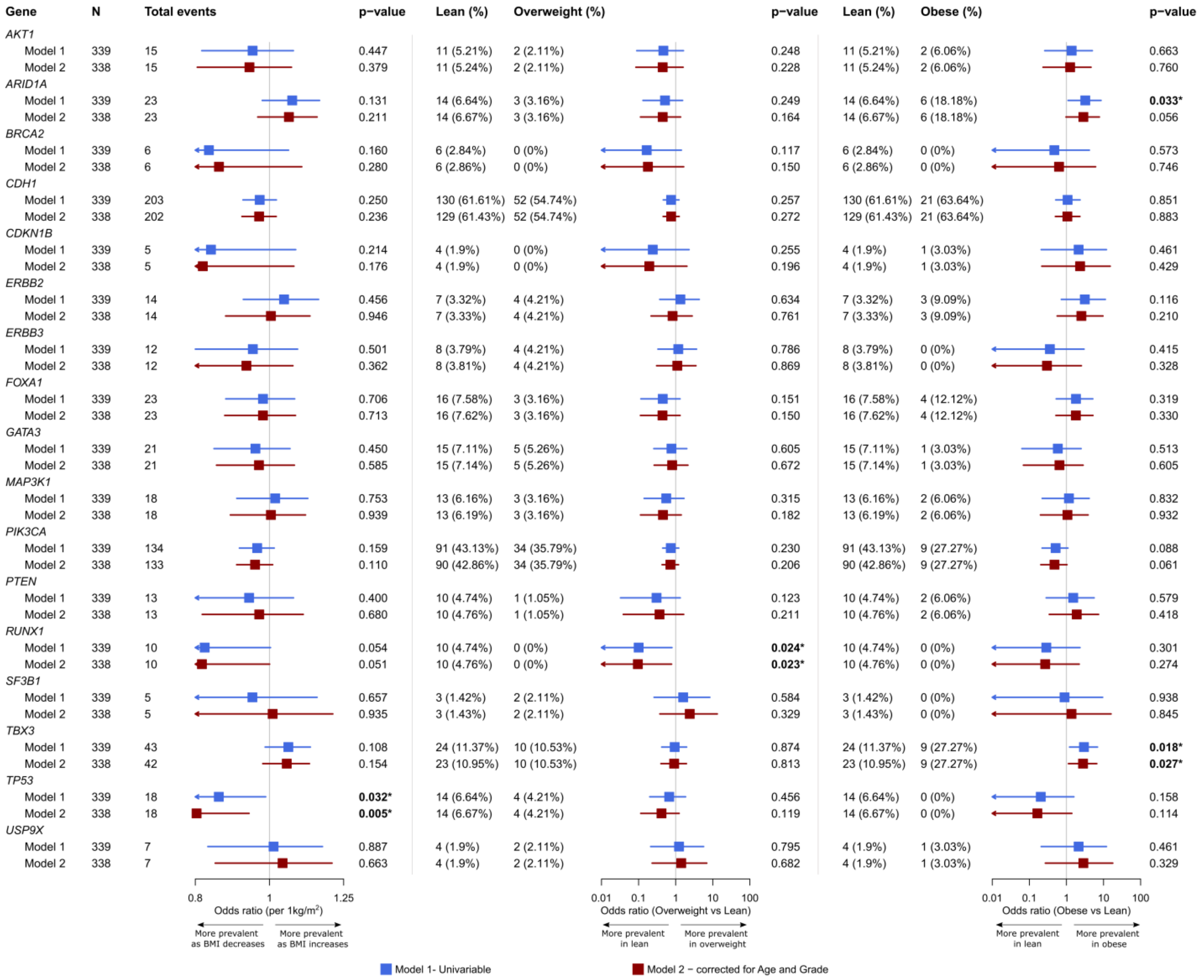

Supplementary Figure 4. Association of BMI with oncogenic mutations of breast cancer-specific driver genes in ILC ER+/HER2- patients. Forest plots showing the associations evaluated using Firth's logistic regression between BMI, as a categorical variable (overweight vs lean, and obese vs lean), and driver gene mutations. Data from the ELBC cohort was used. Gene mutations with at least 5 events detected in the respective cohorts were evaluated. Color-coded boxes indicate point estimates of odds ratios, and whiskers indicate their corresponding 95% confidence intervals. All statistical tests were two-sided. p-values shown were not corrected for multiple testing.

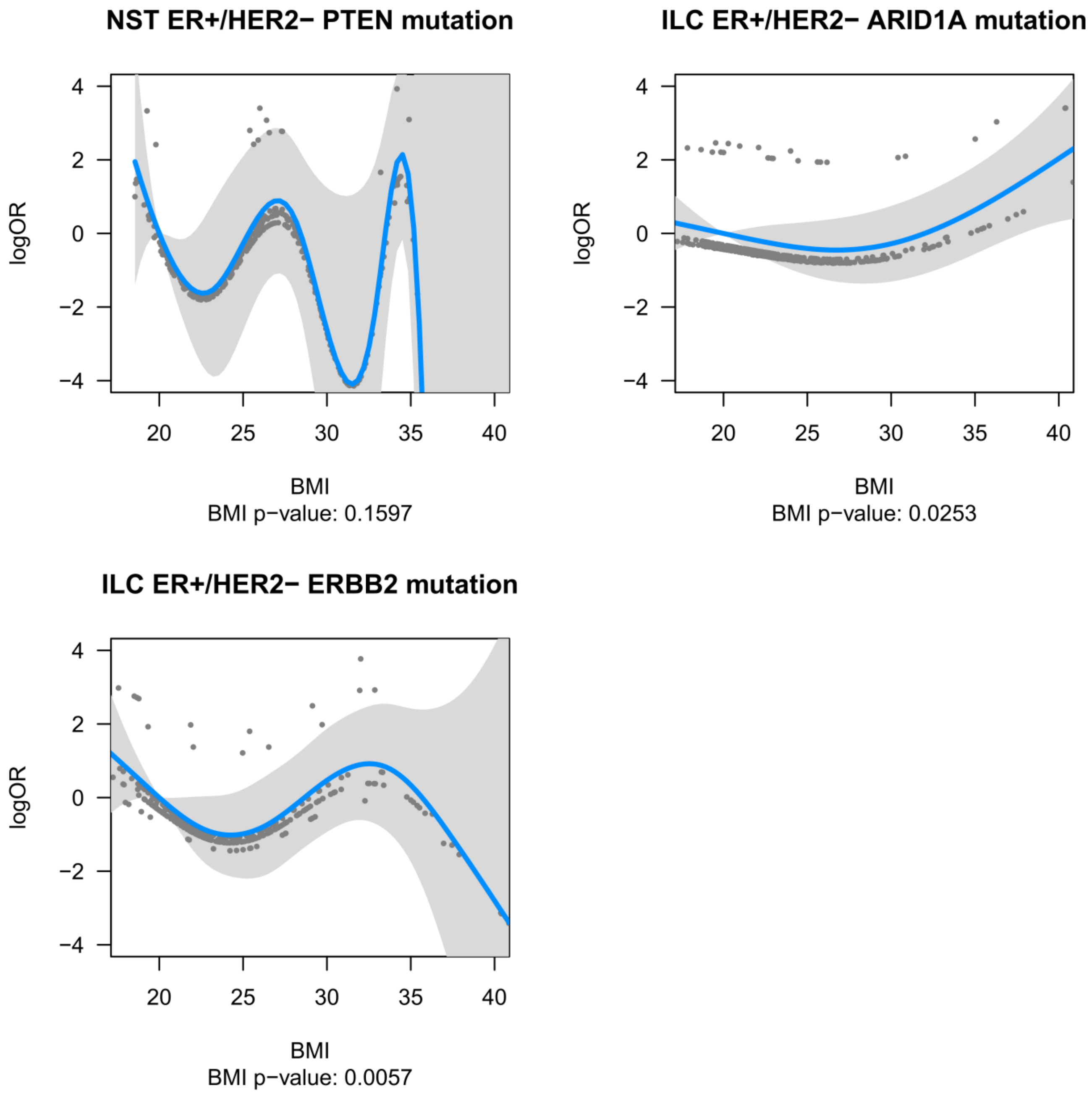

Supplementary Figure 5. Non-linear association of gene mutations with BMI. Plots showing fitted lines of the logOR of gene mutations against continuous BMI (baseline BMI of 20) for those indicated to be better associated with BMI in a non-linear model. Data are presented for point estimates of log-odds ratio (logOR; blue solid line) and their 95% confidence interval (grey shaded band). p-values shown were determined for coefficients estimated by generalized additive models fitted with a spline term of BMI and adjusted for cohort, age, and tumor grade. All statistical tests were two-sided.

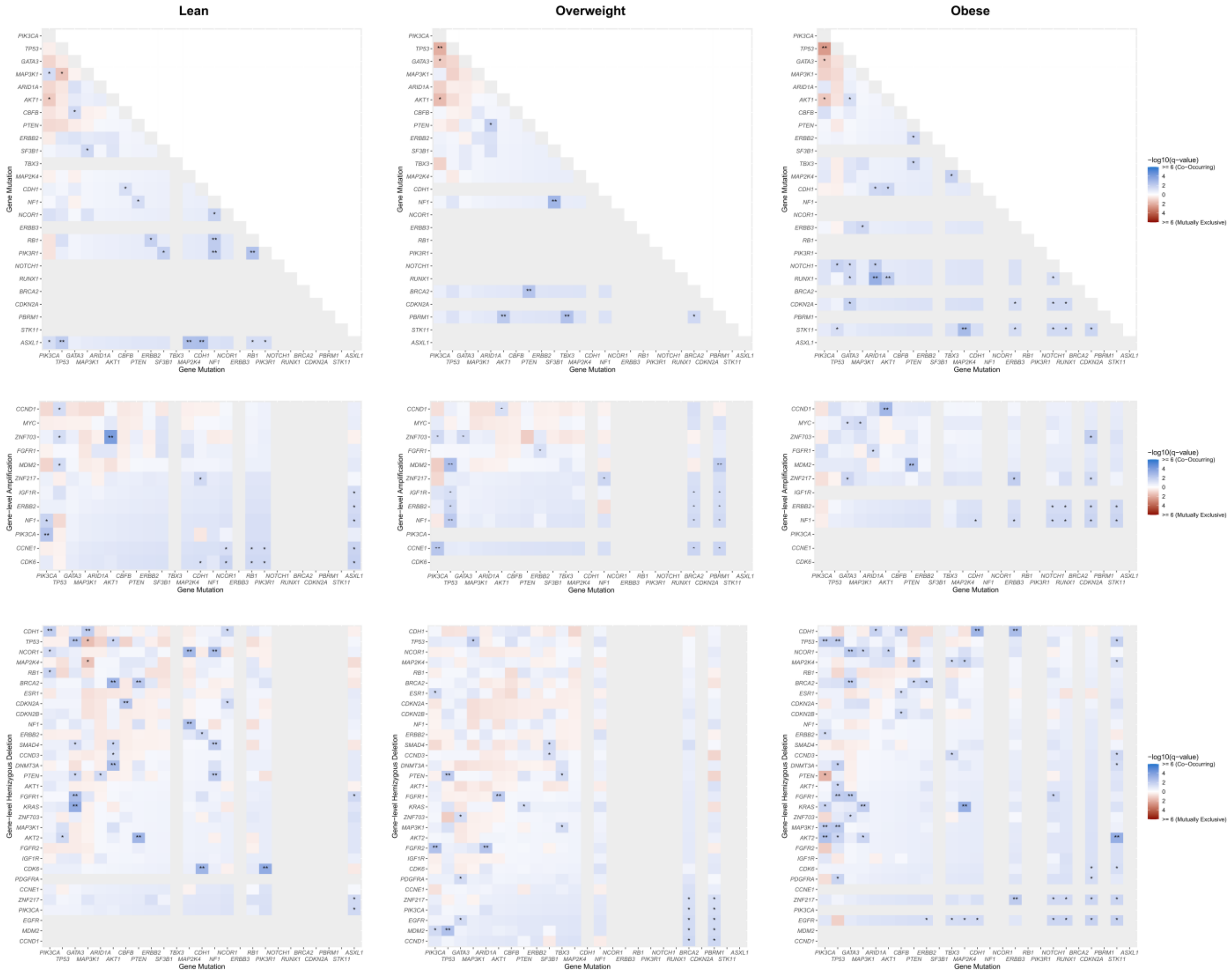

Supplementary Figure 6. Co-occurrence and mutual exclusivity of genomic alterations in NST ER+/HER2- tumors from patients of different BMI categories. Pairwise analyses of recurrent gene mutations and mutations, mutations and CNAs (either amp or hemiLoss) were performed. The color scale represents -log(q-value), which were derived from a Poisson-Binomial distribution-based test and adjusted for multiple testing using the Benjamin-Hochberg method. A grey cell indicates that one of the alterations in the pair did not have enough number of events (3 events) in the respective sub-cohort and thus was not analyzed. Merged data from two cohorts, METABRIC and ICGC, was used.

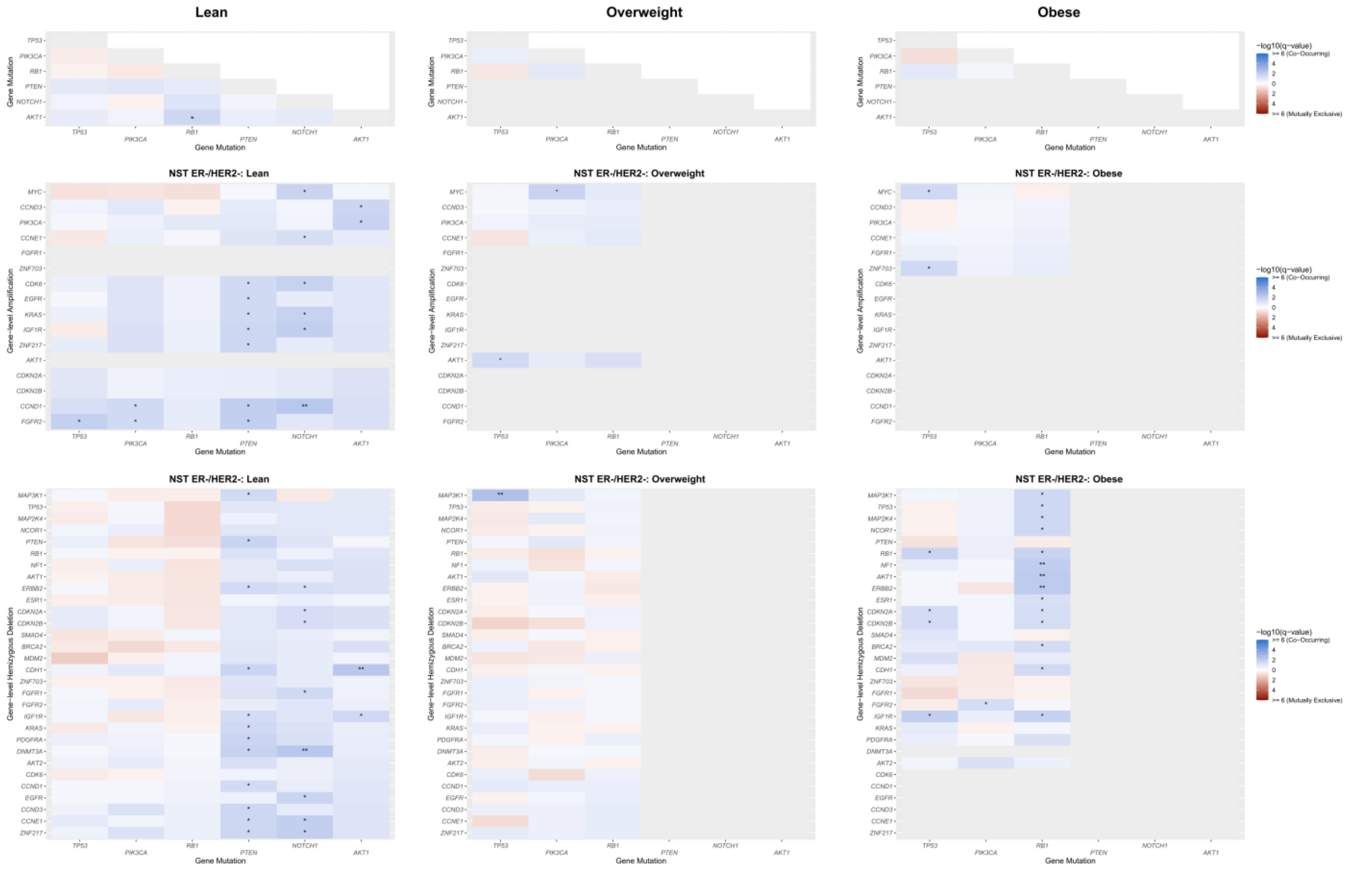

Supplementary Figure 7. Co-occurrence and mutual exclusivity of genomic alterations in NST ER-/HER2- tumors from patients of different BMI categories. Pairwise analyses of recurrent gene mutations and mutations, mutations and CNAs (either amp or hemiLoss) were performed. The color scale represents -log(q-value), which were derived from a Poisson-Binomial distribution-based test and adjusted for multiple testing using the Benjamin-Hochberg method. A grey cell indicates that one of the alterations in the pair did not have enough number of events (3 events) in the respective sub-cohort and thus was not analyzed. Merged data from two cohorts, METABRIC and ICGC, was used.

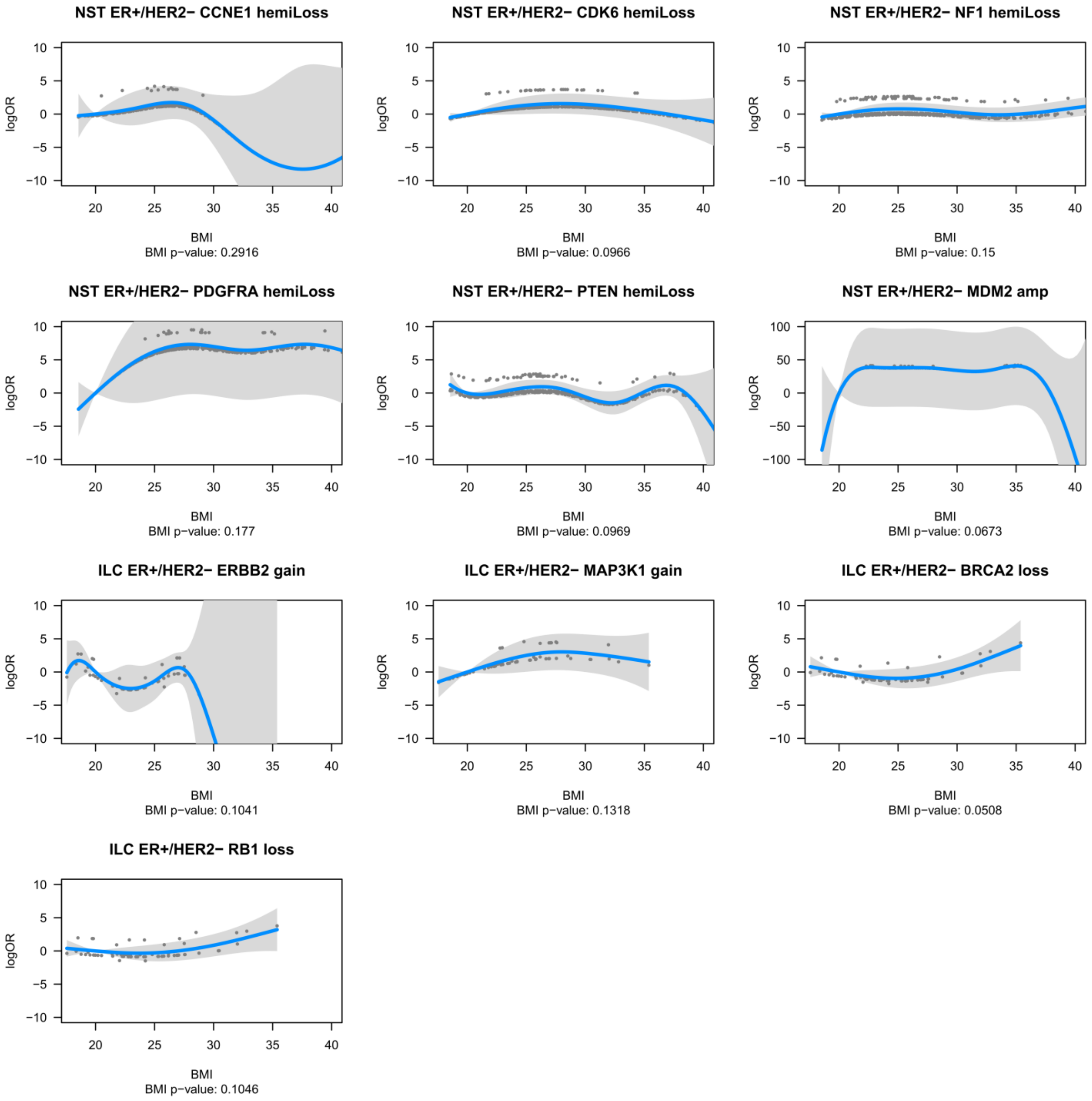

Supplementary Figure 8. Non-linear association of gene-level CNAs with BMI. Plots showing fitted lines of the logOR of gene-level CNAs against continuous BMI (baseline BMI of 20) for those indicated to be better associated with BMI in a non-linear model. Data are presented for point estimates of log-odds ratio (logOR; blue solid line) and their 95% confidence interval (grey shaded band). p-values shown were determined for coefficients estimated by generalized additive models fitted with a spline term of BMI and adjusted for cohort, age, and tumor grade. All statistical tests were two-sided.

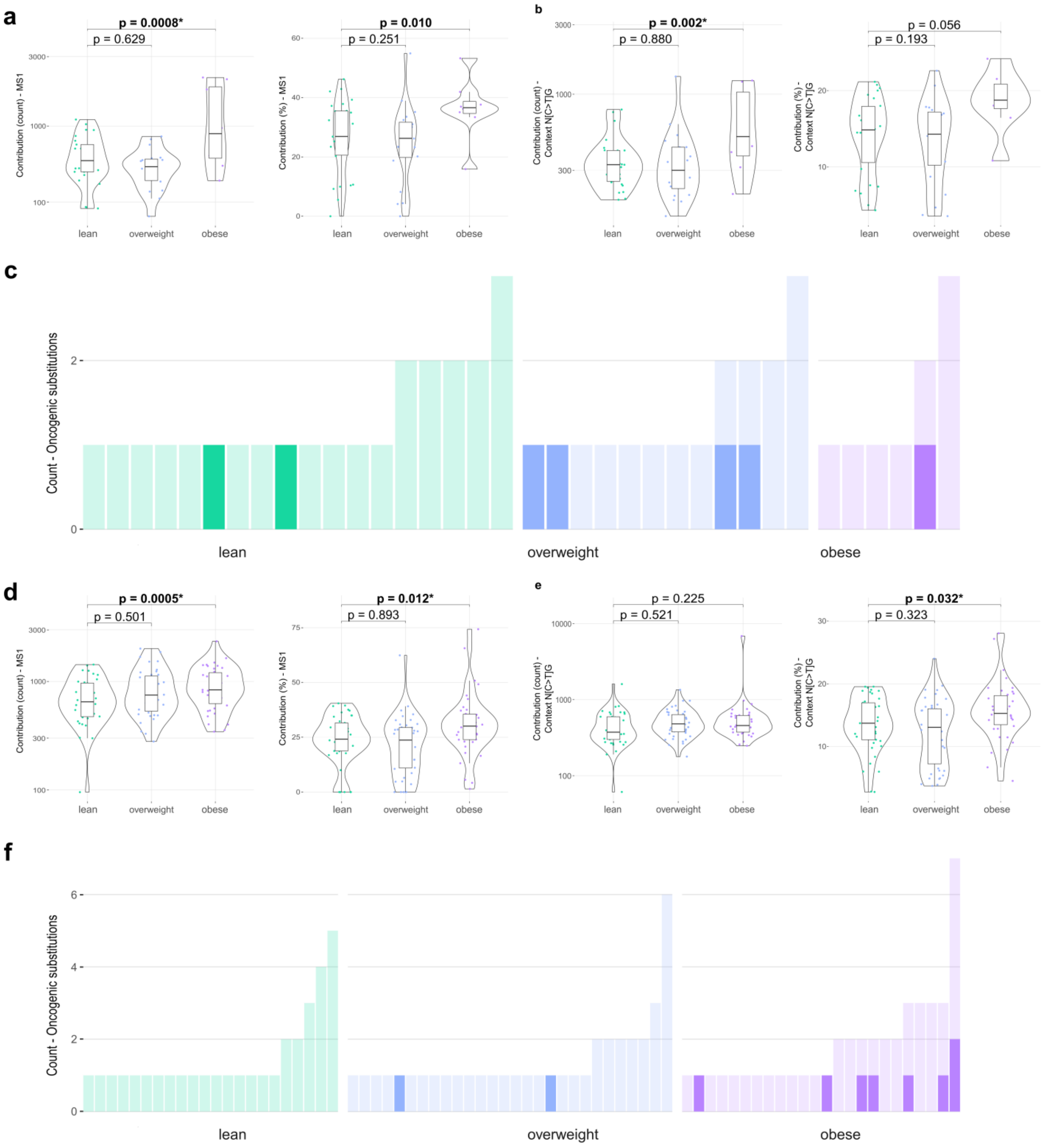

Supplementary Figure 9. Association of BMI with the age-associated mutational signature (Signature.1) in patients with NST ER+/HER2- in different age groups, ≤50 (a-c) and >50 years (d-f), from the ICGC cohort. **a-b, d-e** Violin/box plots showing the contribution in absolute count (left) and relative percentage (right) of the mutational signature 1 (MS1) (a, d) and the sequence context N[C>T]G (b, e) according to BMI categories (age ≤50 lean, n = 26; overweight, n = 20; obese, n = 8; age >50 lean, n = 41; overweight, n = 44; obese, n = 38). In each boxplot, the box denotes the range from the 25th to the 75th percentile, the center line indicates the median value, and the whiskers specify the

maxima and minima excluding outliers. All statistical tests were two-sided. Wald test p-values determined for coefficients estimated by linear regressions adjusted for age continuous) and tumor grade (G3 vs G1/G2), are reported and were not corrected for multiple testing. **c, f** Bar plots presenting the number of oncogenic mutations occurring in BC-specific driver genes in each tumor. The count of mutations having the sequence context N[C>T]G is highlighted in bold colors. Each bar represents a tumor.

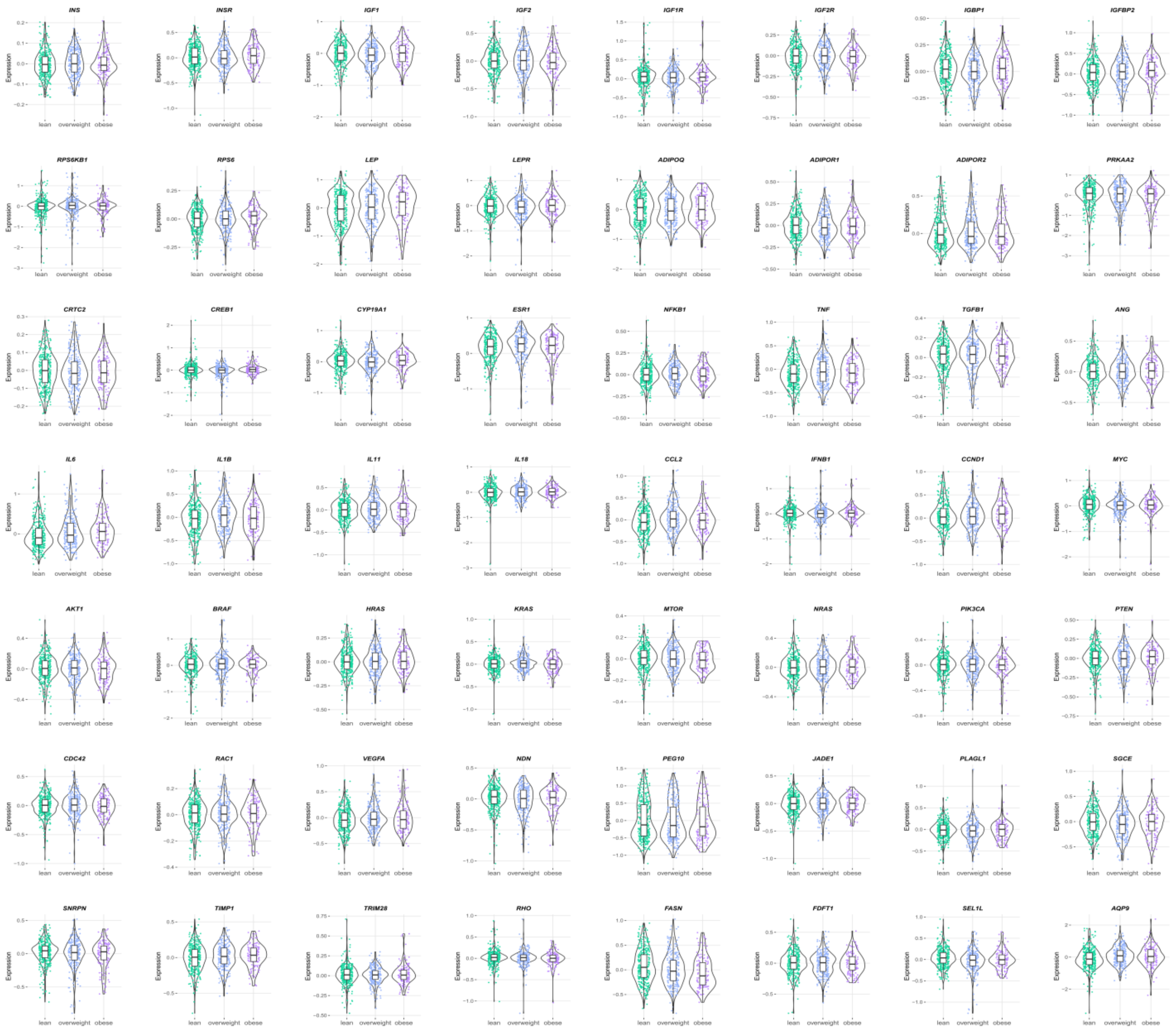

Supplementary Figure 10. Gene expression of relevant genes in the BC-obesity axis in NST ER+/HER2- tumors from patients of different BMI categories in the MINDACT cohort. Violin/box plots showing the expression levels of selected genes according to BMI categories (lean, n = 354; overweight, n = 250; obese, n = 131). The expression values (log10-transformed) presented here had been normalized prior to data retrieval. In each boxplot, the box denotes the range from the 25th to the 75th percentile, the center line indicates the median value, and the whiskers specify the maxima and minima excluding outliers.

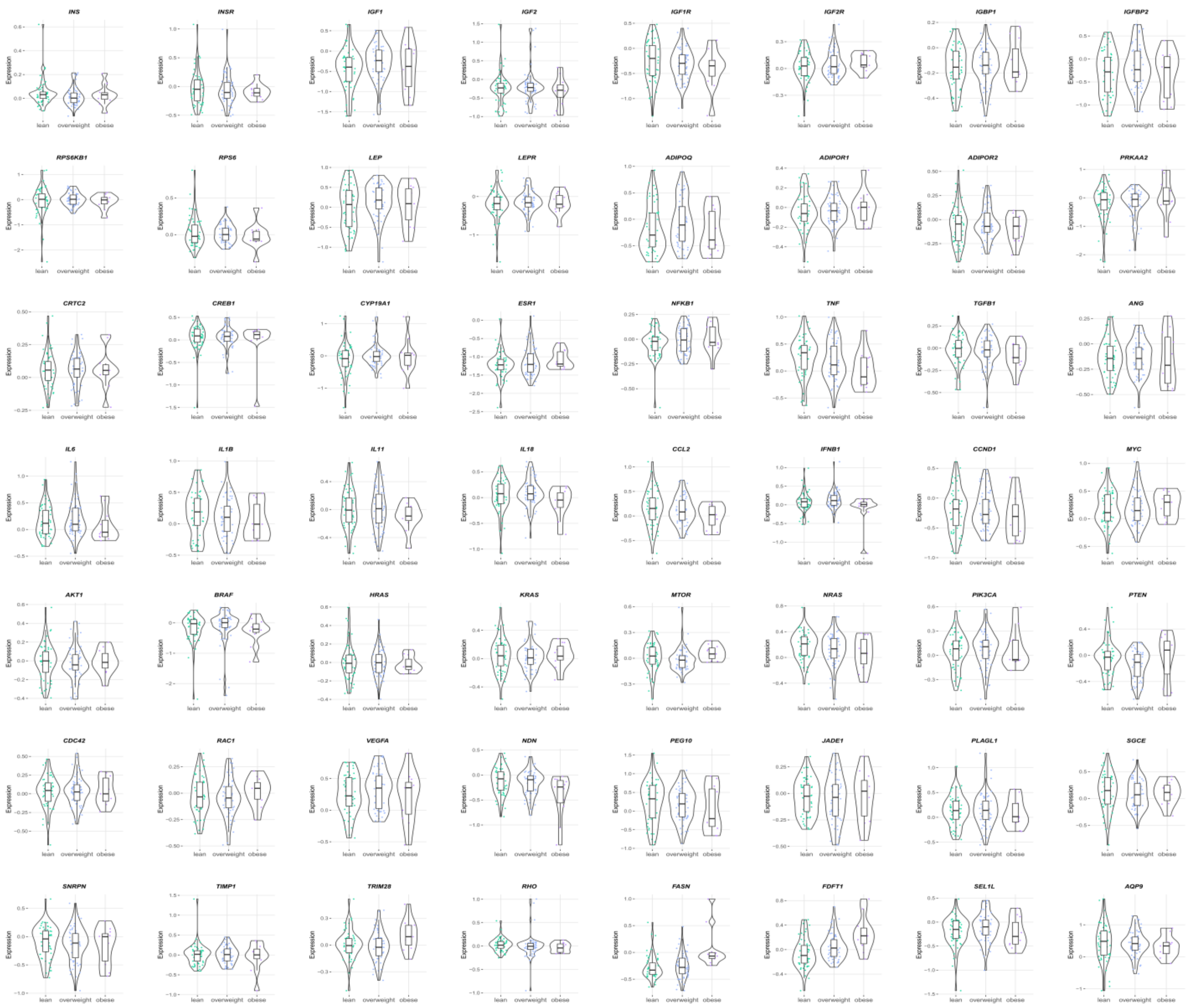

Supplementary Figure 11. Gene expression of relevant genes in the BC-obesity axis in NST ER-/HER2- tumors from patients of different BMI categories in the MINDACT cohort. Violin/box plots showing the expression levels of selected genes according to BMI categories (lean, n = 53; overweight, n = 54; obese, n = 11). The expression values (log10-transformed) presented here had been normalized prior to data retrieval. In each boxplot, the box denotes the range from the 25th to the 75th percentile, the center line indicates the median value, and the whiskers specify the maxima and minima excluding outliers.

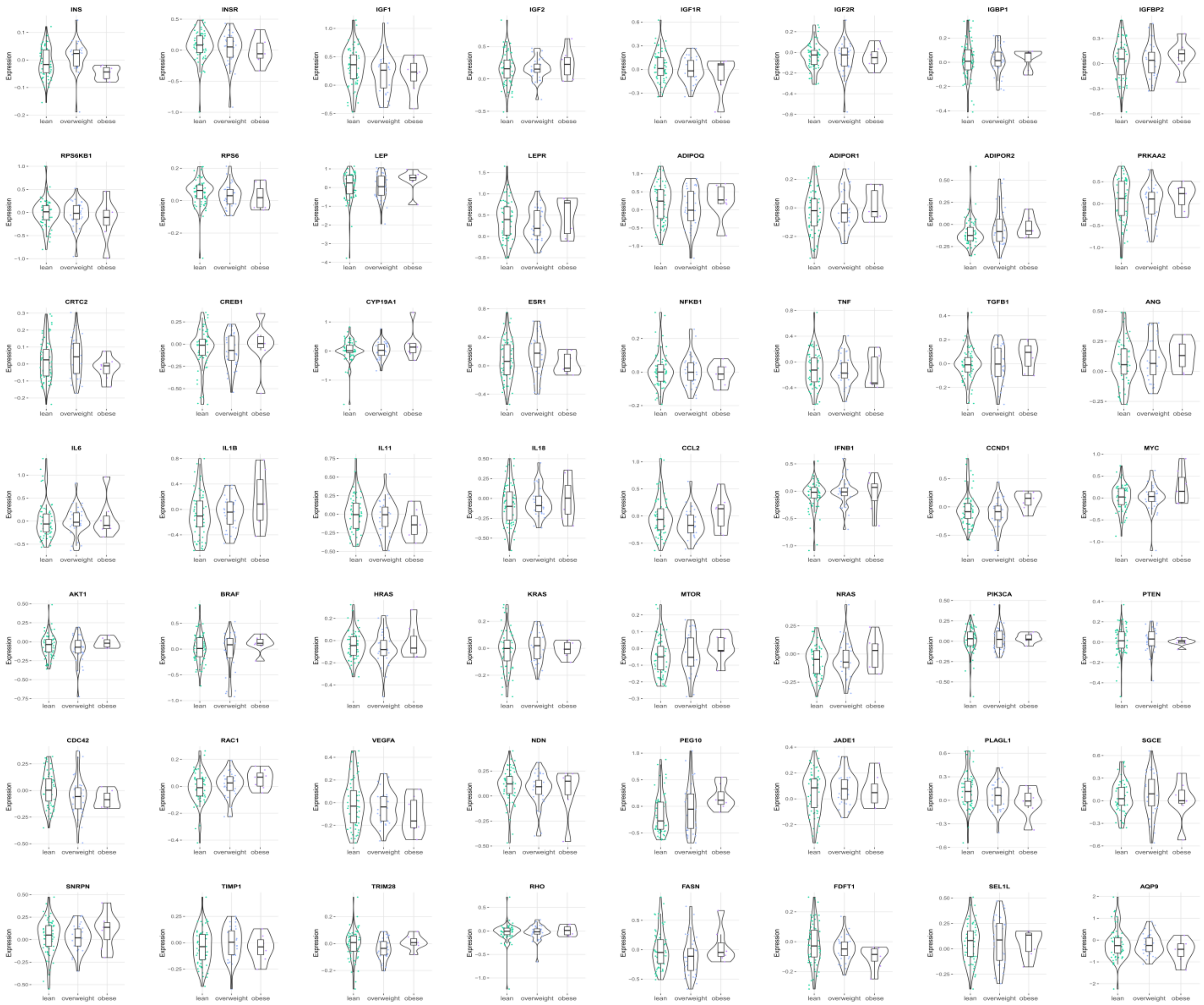

Supplementary Figure 12. Gene expression of relevant genes in the BC-obesity axis in ILC ER+/HER2- tumors from patients of different BMI categories in the MINDACT cohort. Violin/box plots showing the expression levels of selected genes according to BMI categories (lean, n = 65; overweight, n = 32; obese, n = 7). The expression values (log10-transformed) presented here had been normalized prior to data retrieval. In each boxplot, the box denotes the range from the 25th to the 75th percentile, the center line indicates the median value, and the whiskers specify the maxima and minima excluding outliers.

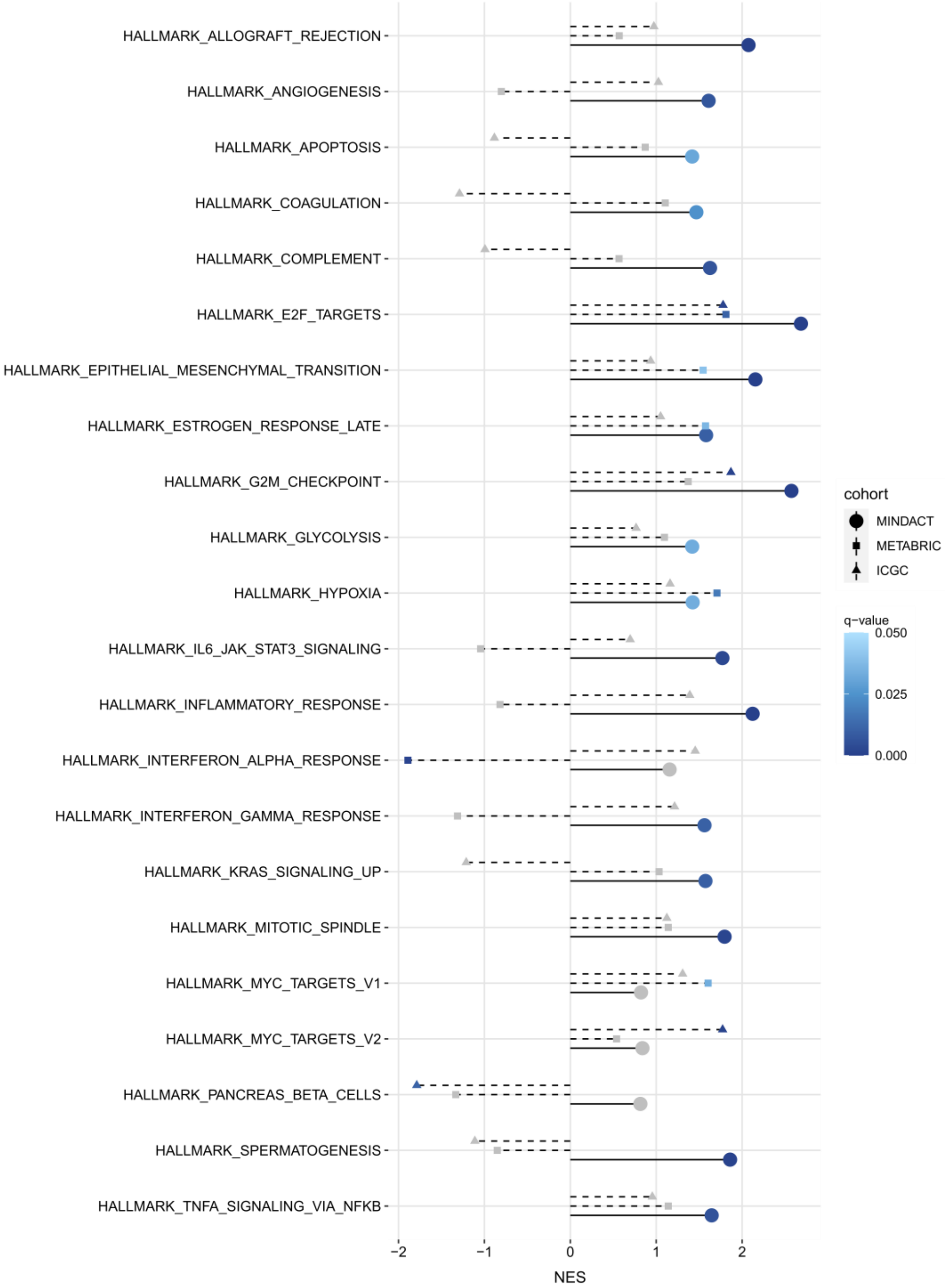

Supplementary Figure 13. Differentially enriched hallmarks in NST ER+/HER2- tumors from obese patients compared to lean patients. Lollipop plots displaying differentially enriched molecular hallmarks detected by GSEA (q-value < 0.05) in at least one of the analyzed cohorts. The signs of the normalized enrichment score (NES) indicate the orientation of the differential enrichments (positive: enriched in tumors from obese patients, negative: enriched in tumors from lean patients). Lollipops with solid segments represent the main cohort discussed in the main text (i.e. MINDACT) and those with dashed segments represent the other cohorts.

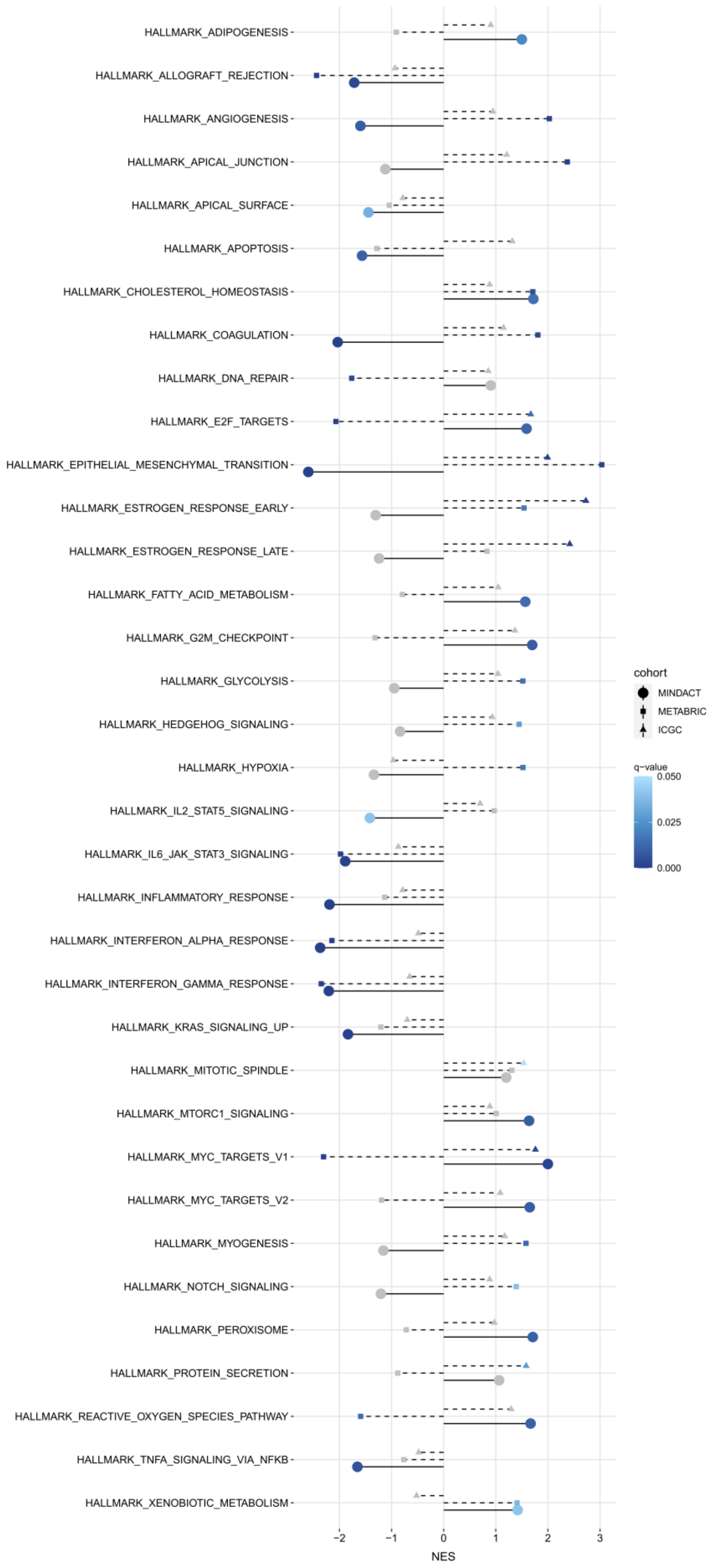

HALLMARK_ADIPOGENESIS
HALLMARK_ALLOGRAFT_REJECTION
HALLMARK_ANGIOGENESIS
HALLMARK_APICAL_JUNCTION
HALLMARK_APICAL_SURFACE
HALLMARK_APOPTOSIS
HALLMARK_CHOLESTEROL_HOMEOSTASIS
HALLMARK_COAGULATION
HALLMARK_DNA_REPAIR
HALLMARK_E2F_TARGETS
HALLMARK_EPITHELIAL_MESENCHYMAL_TRANSITION
HALLMARK_ESTROGEN_RESPONSE_EARLY
HALLMARK_ESTROGEN_RESPONSE_LATE
HALLMARK_FATTY_ACID_METABOLISM
HALLMARK_G2M_CHECKPOINT
HALLMARK_GLYCOLYSIS
HALLMARK_HEDGEHOG_SIGNALING
HALLMARK_HYPOXIA
HALLMARK_IL2_STAT5_SIGNALING
HALLMARK_IL6_JAK_STAT3_SIGNALING
HALLMARK_INFLAMMATORY_RESPONSE
HALLMARK_INTERFERON_ALPHA_RESPONSE
HALLMARK_INTERFERON_GAMMA_RESPONSE
HALLMARK_KRAS_SIGNALING_UP
HALLMARK_MITOTIC_SPINDLE
HALLMARK_MTORC1_SIGNALING
HALLMARK_MYC_TARGETS_V1
HALLMARK_MYC_TARGETS_V2
HALLMARK_MYOGENESIS
HALLMARK_NOTCH_SIGNALING
HALLMARK_PEROXISOME
HALLMARK_PROTEIN_SECRETION
HALLMARK_REACTIVE_OXYGEN_SPECIES_PATHWAY
HALLMARK_TNFA_SIGNALING_VIA_NFKB
HALLMARK_XENOBIOTIC_METABOLISM
−2
−1
0
1
2
3
NES
cohort
MINDACT
METABRIC
ICGC
q−value
0.050
0.025
0.000

Supplementary Figure 14. Differentially enriched hallmarks in NST ER-/HER2- tumors from obese patients compared to lean patients. Lollipop plots displaying differentially enriched molecular hallmarks detected by GSEA (q-value < 0.05) in at least one of the analyzed cohorts. The signs of the normalized enrichment score (NES) indicate the orientation of the differential enrichments (positive: enriched in tumors from obese patients, negative: enriched in tumors from lean patients). Lollipops with solid segments represent the main cohort discussed in the main text (i.e. MINDACT) and those with dashed segments represent the other cohorts.

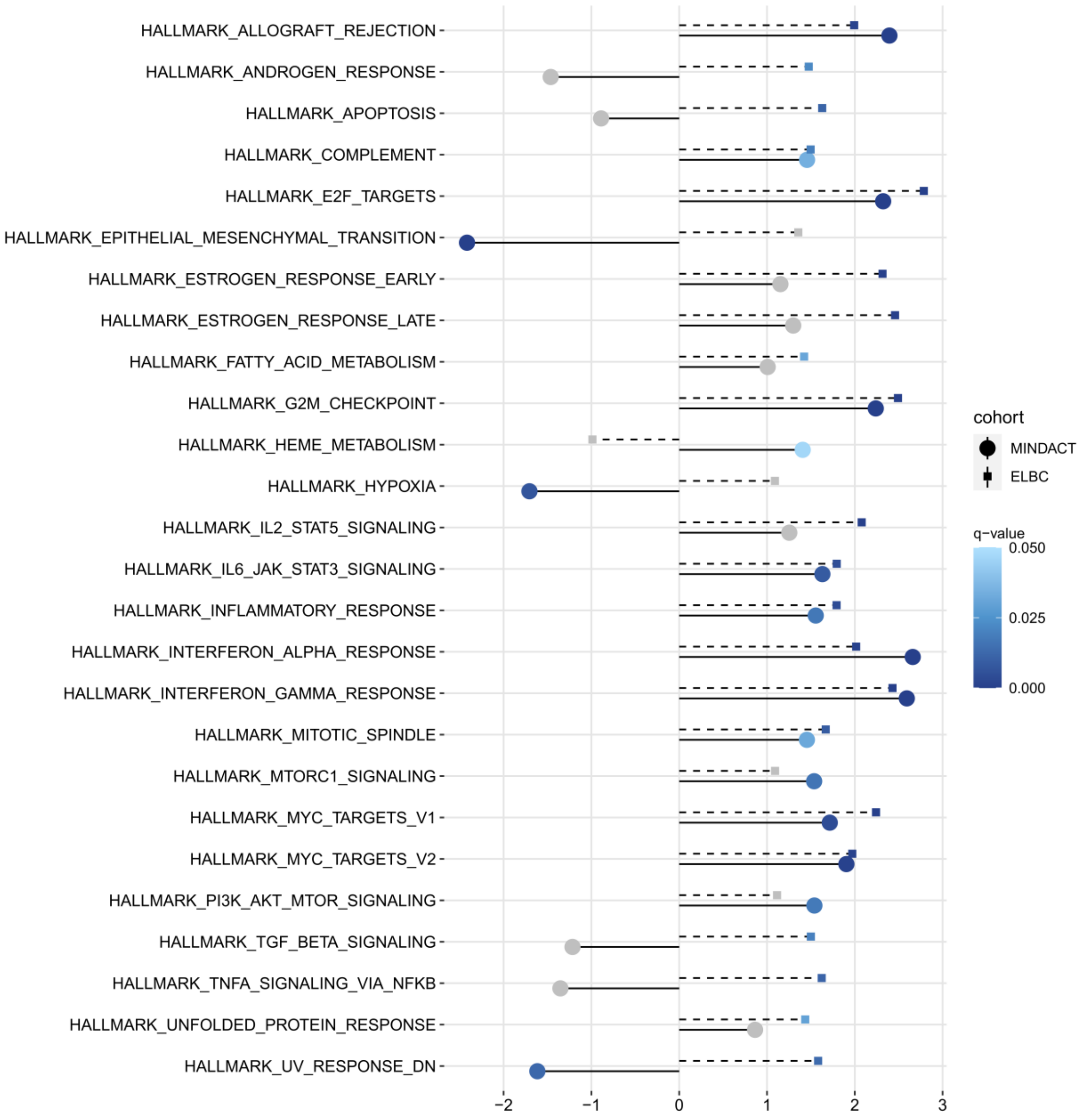

Supplementary Figure 15. Differentially enriched hallmarks in ILC ER+/HER2- tumors from obese patients compared to lean patients. Lollipop plots displaying differentially enriched molecular hallmarks detected by GSEA (q-value < 0.05) in at least one of the analyzed cohorts. The signs of the normalized enrichment score (NES) indicate the orientation of the differential enrichments (positive: enriched in tumors from obese patients, negative: enriched in tumors from lean patients). Lollipops with solid segments represent the main cohort discussed in the main text (i.e. MINDACT) and those with dashed segments represent the other cohorts.

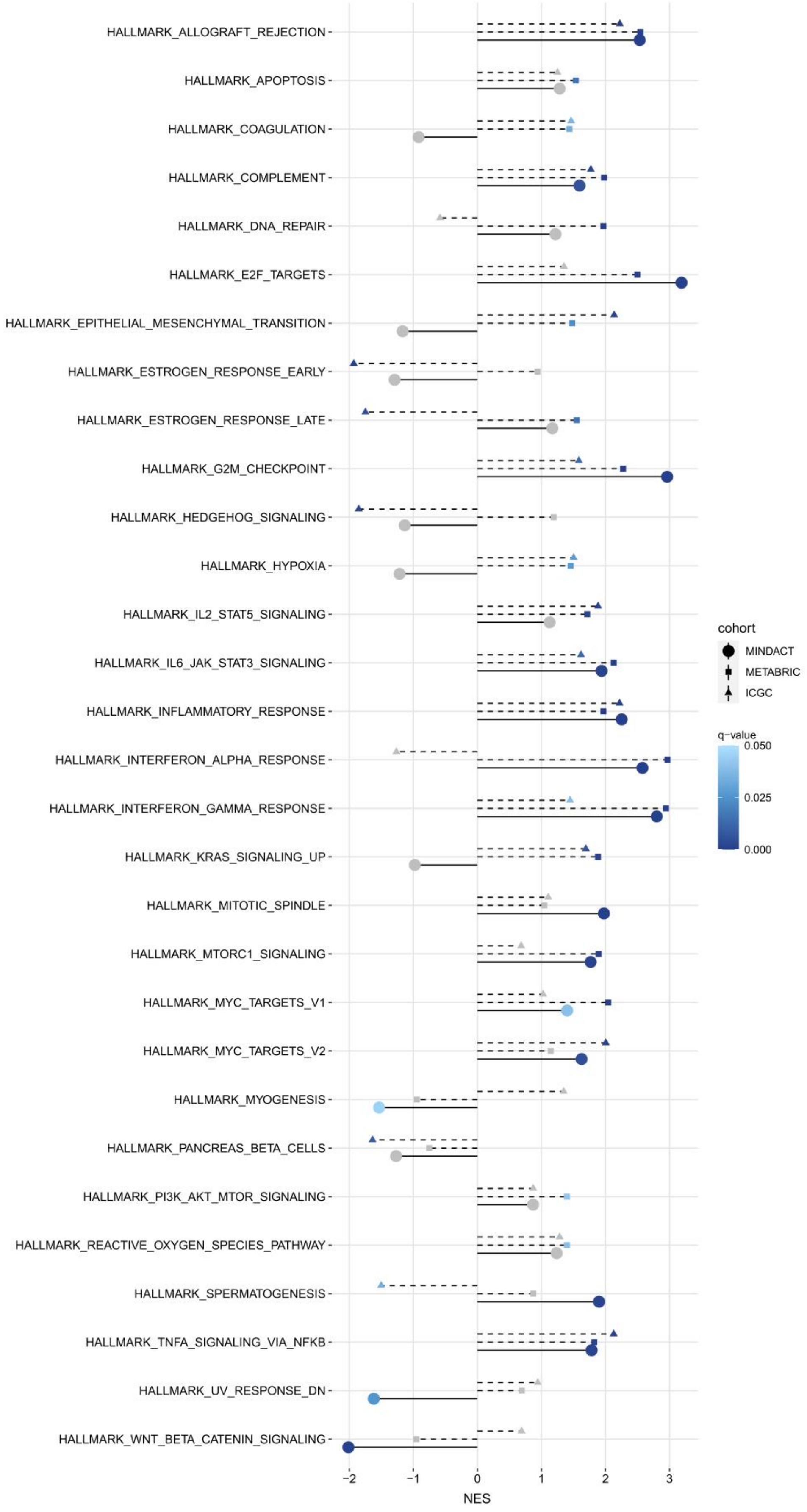
HALLMARK_ALLOGRAFT_REJECTION
HALLMARK_APOPTOSIS
HALLMARK_COAGULATION
HALLMARK_COMPLEMENT
HALLMARK_DNA_REPAIR
HALLMARK_E2F_TARGETS
HALLMARK_EPITHELIAL_MESENCHYMAL_TRANSITION
HALLMARK_ESTROGEN_RESPONSE_EARLY
HALLMARK_ESTROGEN_RESPONSE_LATE
HALLMARK_G2M_CHECKPOINT
HALLMARK_HEDGEHOG_SIGNALING
HALLMARK_HYPOXIA
HALLMARK_IL2_STAT5_SIGNALING
HALLMARK_IL6_JAK_STAT3_SIGNALING
HALLMARK_INFLAMMATORY_RESPONSE
HALLMARK_INTERFERON_ALPHA_RESPONSE
HALLMARK_INTERFERON_GAMMA_RESPONSE
HALLMARK_KRAS_SIGNALING_UP
HALLMARK_MITOTIC_SPINDLE
HALLMARK_MTORC1_SIGNALING
HALLMARK_MYC_TARGETS_V1
HALLMARK_MYC_TARGETS_V2
HALLMARK_MYOGENESIS
HALLMARK_PANCREAS_BETA_CELLS
HALLMARK_PI3K_AKT_MTOR_SIGNALING
HALLMARK_REACTIVE_OXYGEN_SPECIES_PATHWAY
HALLMARK_SPERMATOGENESIS
HALLMARK_TNFA_SIGNALING_VIA_NFKB
HALLMARK_UV_RESPONSE_DN
HALLMARK_WNT_BETA_CATENIN_SIGNALING
−2
−1
0
1
2
3
NES
cohort
MINDACT
METABRIC
ICGC
q−value
0.050
0.025
0.000

Supplementary Figure 16. Differentially enriched hallmarks in NST ER+/HER2- tumors from overweight patients compared to lean patients. Lollipop plots displaying differentially enriched molecular hallmarks detected by GSEA (q-value < 0.05) in at least one of the analyzed cohorts. The signs of the normalized enrichment score (NES) indicate the orientation of the differential enrichments (positive: enriched in tumors from overweight patients, negative: enriched in tumors from lean patients). Lollipops with solid segments represent the main cohort discussed in the main text (i.e. MINDACT) and those with dashed segments represent the other cohorts.

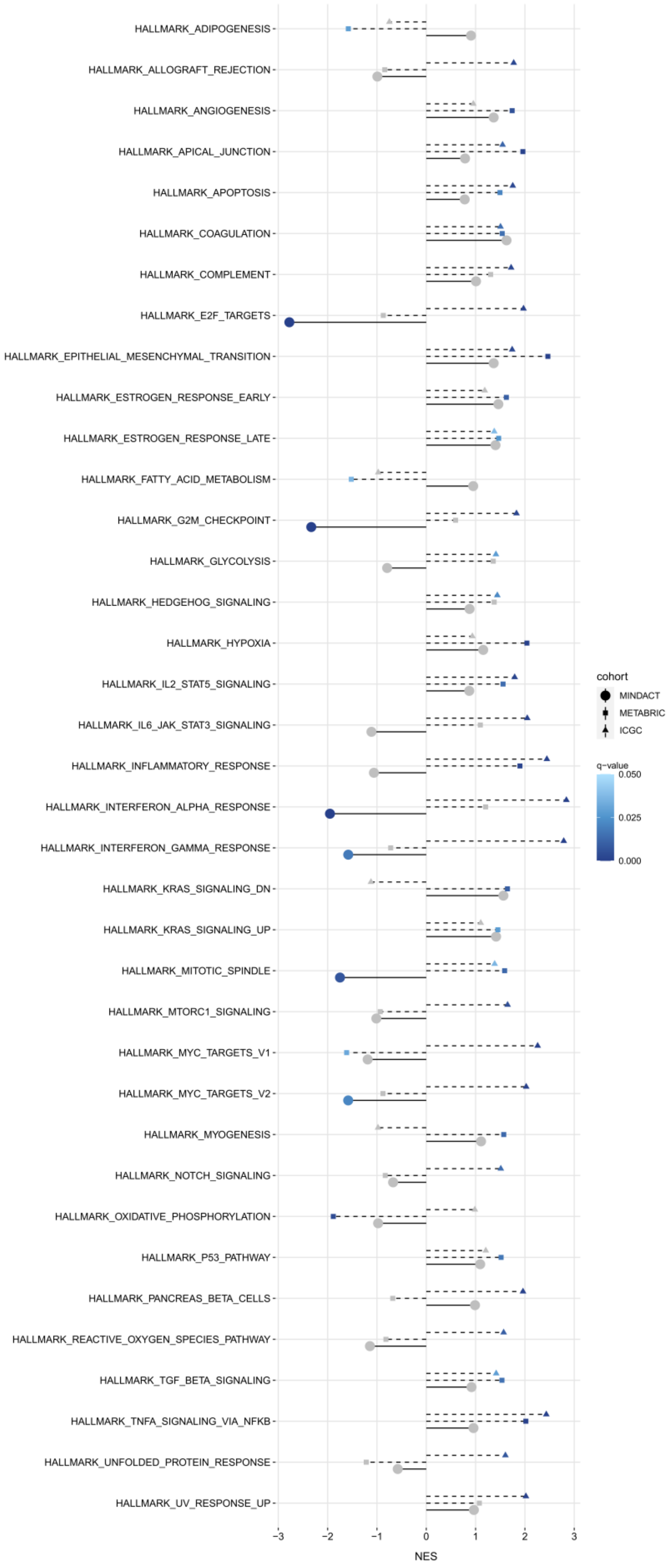

HALLMARK_ADIPOGENESIS
HALLMARK_ALLOGRAFT_REJECTION
HALLMARK_ANGIOGENESIS
HALLMARK_APICAL_JUNCTION
HALLMARK_APOPTOSIS
HALLMARK_COAGULATION
HALLMARK_COMPLEMENT
HALLMARK_E2F_TARGETS
HALLMARK_EPITHELIAL_MESENCHYMAL_TRANSITION
HALLMARK_ESTROGEN_RESPONSE_EARLY
HALLMARK_ESTROGEN_RESPONSE_LATE
HALLMARK_FATTY_ACID_METABOLISM
HALLMARK_G2M_CHECKPOINT
HALLMARK_GLYCOLYSIS
HALLMARK_HEDGEHOG_SIGNALING
HALLMARK_HYPOXIA
HALLMARK_IL2_STAT5_SIGNALING
HALLMARK_IL6_JAK_STAT3_SIGNALING
HALLMARK_INFLAMMATORY_RESPONSE
HALLMARK_INTERFERON_ALPHA_RESPONSE
HALLMARK_INTERFERON_GAMMA_RESPONSE
HALLMARK_KRAS_SIGNALING_DN
HALLMARK_KRAS_SIGNALING_UP
HALLMARK_MITOTIC_SPINDLE
HALLMARK_MTORC1_SIGNALING
HALLMARK_MYC_TARGETS_V1
HALLMARK_MYC_TARGETS_V2
HALLMARK_MYOGENESIS
HALLMARK_NOTCH_SIGNALING
HALLMARK_OXIDATIVE_PHOSPHORYLATION
HALLMARK_P53_PATHWAY
HALLMARK_PANCREAS_BETA_CELLS
HALLMARK_REACTIVE_OXYGEN_SPECIES_PATHWAY
HALLMARK_TGF_BETA_SIGNALING
HALLMARK_TNFA_SIGNALING_VIA_NFKB
HALLMARK_UNFOLDED_PROTEIN_RESPONSE
HALLMARK_UV_RESPONSE_UP
−3
−2
−1
0
1
2
3
NES
cohort
MINDACT
METABRIC
ICGC
q−value
0.050
0.025
0.000

Supplementary Figure 17. Differentially enriched hallmarks in NST ER-/HER2- tumors from overweight patients compared to lean patients. Lollipop plots displaying differentially enriched molecular hallmarks detected by GSEA (q-value < 0.05) in at least one of the analyzed cohorts. The signs of the normalized enrichment score (NES) indicate the orientation of the differential enrichments (positive: enriched in tumors from overweight patients, negative: enriched in tumors from lean patients). Lollipops with solid segments represent the main cohort discussed in the main text (i.e. MINDACT) and those with dashed segments represent the other cohorts.

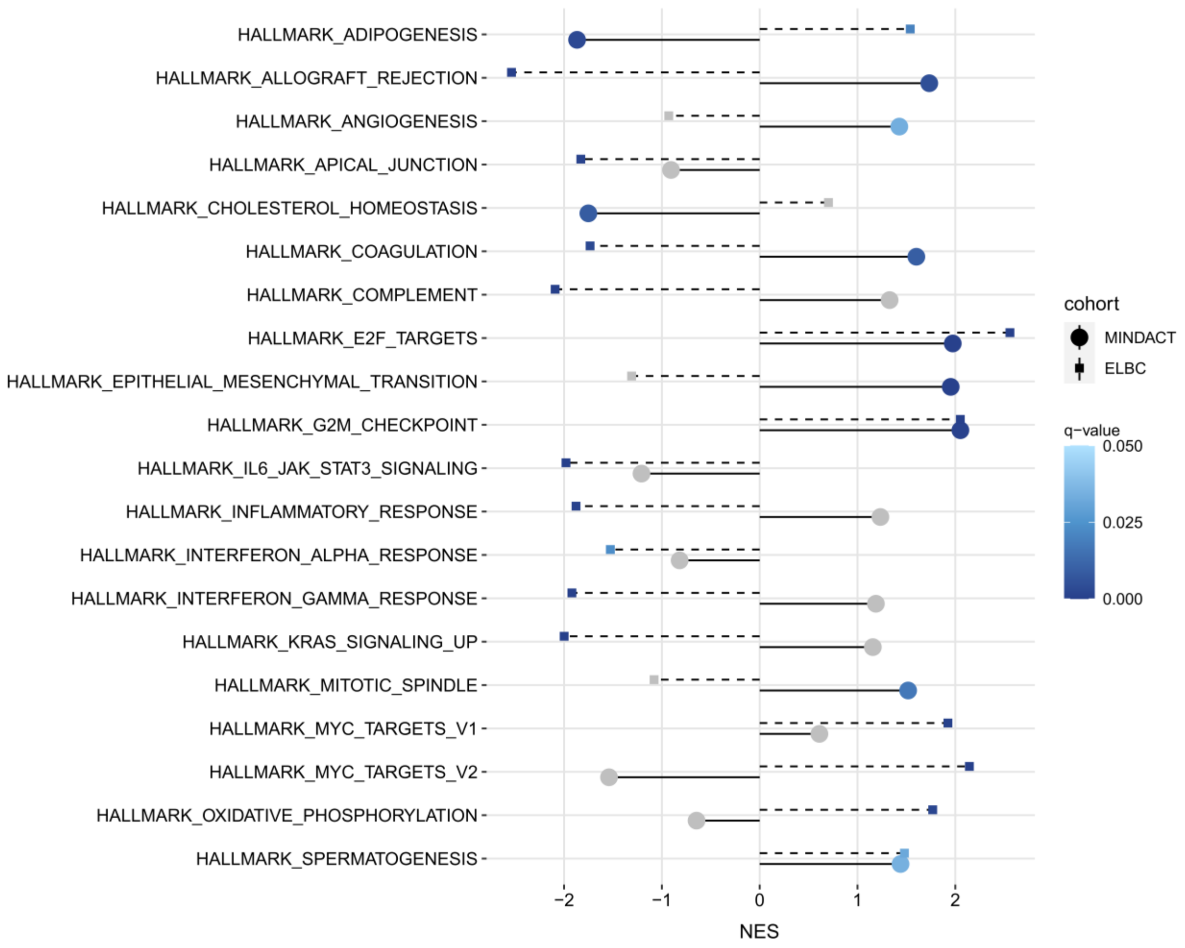

Supplementary Figure 18. Differentially enriched hallmarks in ILC ER+/HER2- tumors from overweight patients compared to lean patients. Lollipop plots displaying differentially enriched molecular hallmarks detected by GSEA (q-value < 0.05) in at least one of the analyzed cohorts. The signs of the normalized enrichment score (NES) indicate the orientation of the differential enrichments (positive: enriched in tumors from overweight patients, negative: enriched in tumors from lean patients). Lollipops with solid segments represent the main cohort discussed in the main text (i.e. MINDACT) and those with dashed segments represent the other cohorts.

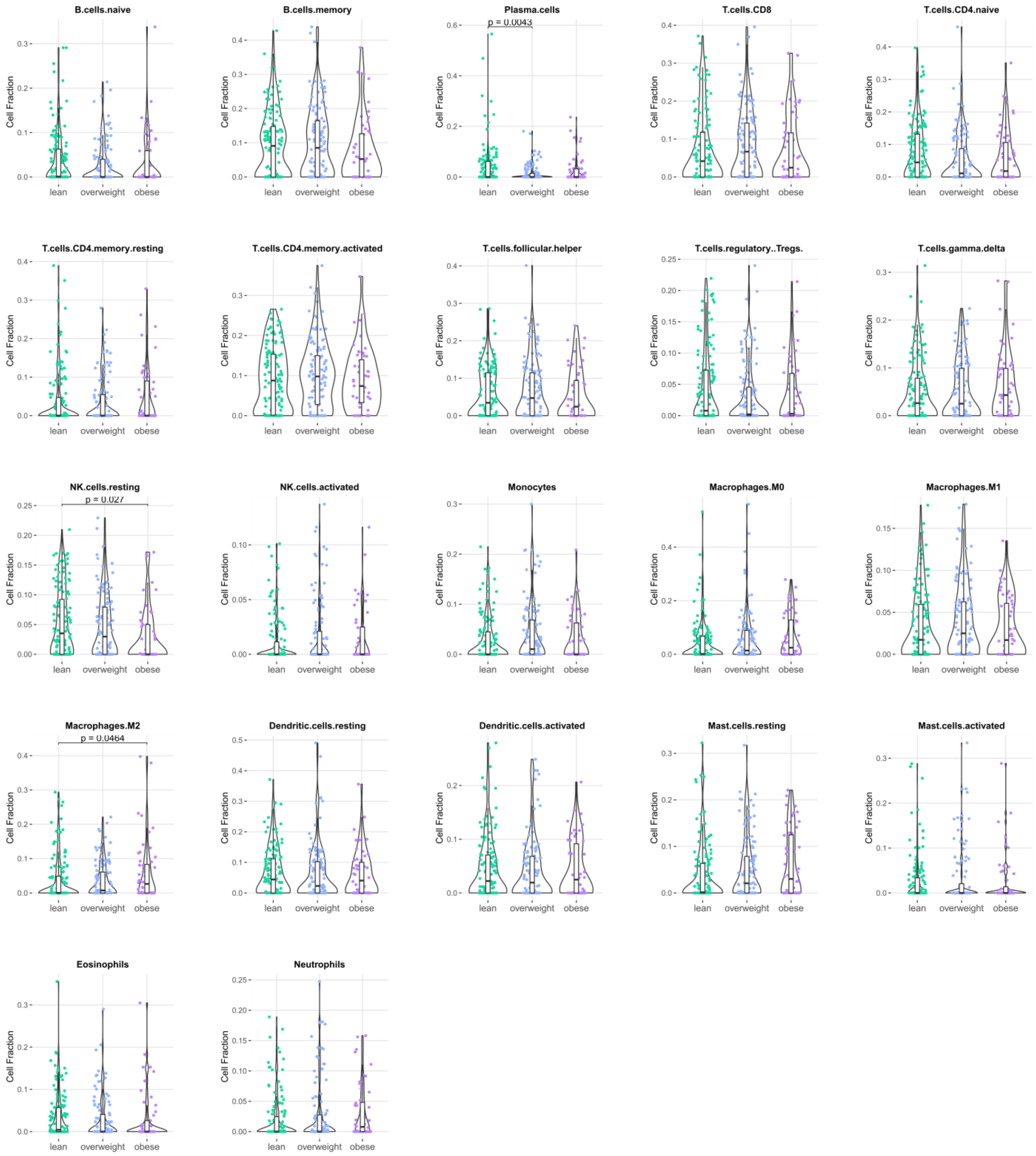

Supplementary Figure 19. Cell fractions of 22 immune cell types in the tumor bulk of NST ER+/HER2- tumors from the MINDACT cohort. Violin/box plots showing relative frequency of 22 immune cells inferred by deconvolution of the bulk profiling data using CIBERSORTx according to BMI categories (lean, n = 354; overweight, n = 250; obese, n = 65). In each boxplot, the box denotes the range from the 25th to the 75th percentile, the center line indicates the median value, and the whiskers specify the maxima and minima excluding outliers. Comparisons with p-value < 0.05 are indicated. p-values shown were derived from Wilcoxon's ranked sum tests. All statistical tests were two-sided.

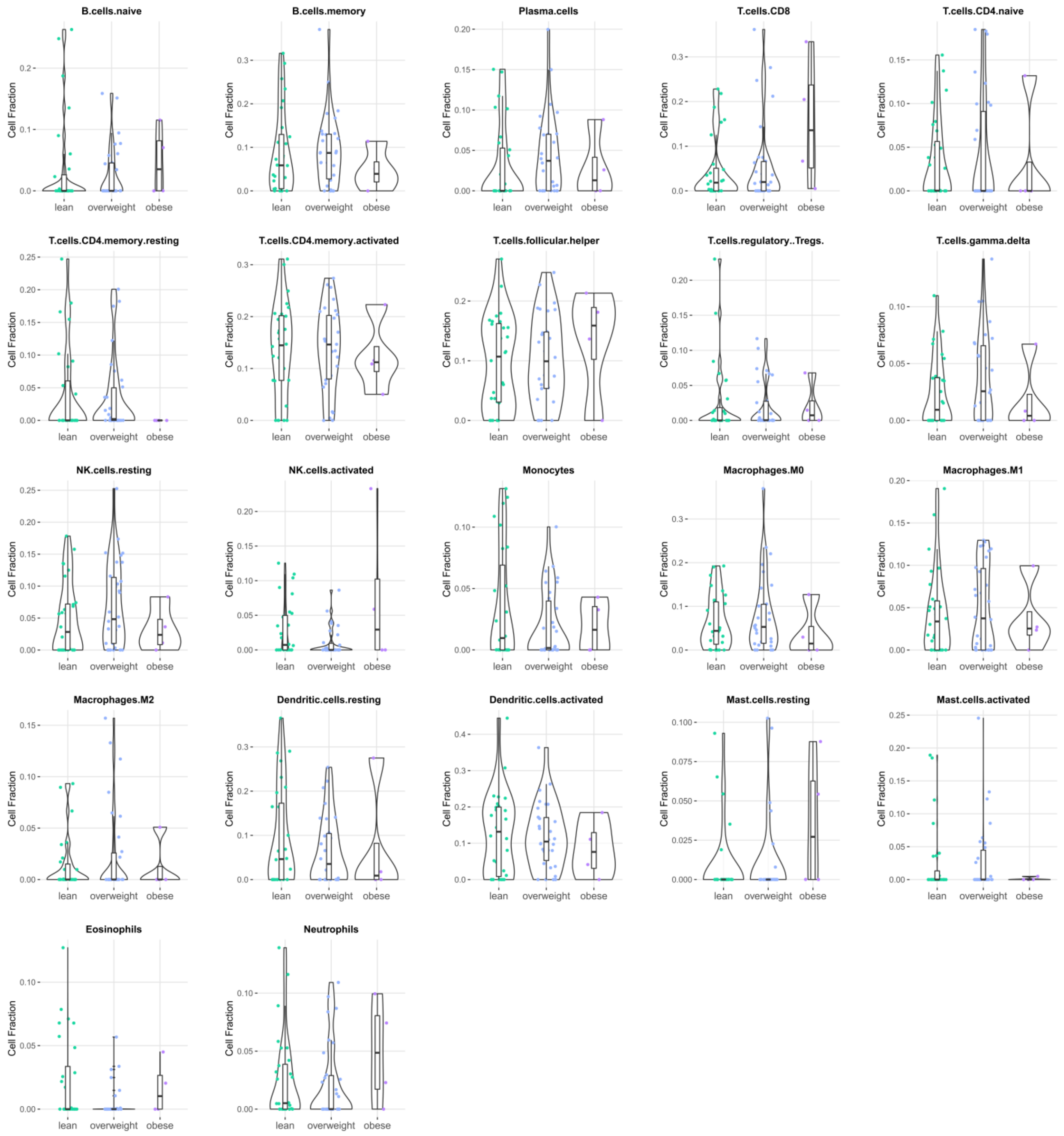

Supplementary Figure 20. Cell fractions of 22 immune cell types in the tumor bulk of NST ER-/HER2- tumors from the MINDACT cohort. Violin plots showing relative frequency of 22 immune cells inferred by deconvolution of the bulk profiling data using CIBERSORTx according to BMI categories (lean, n = 53; overweight, n = 54; obese, n = 11). In each boxplot, the box denotes the range from the 25th to the 75th percentile, the center line indicates the median value, and the whiskers specify the maxima and minima excluding outliers.

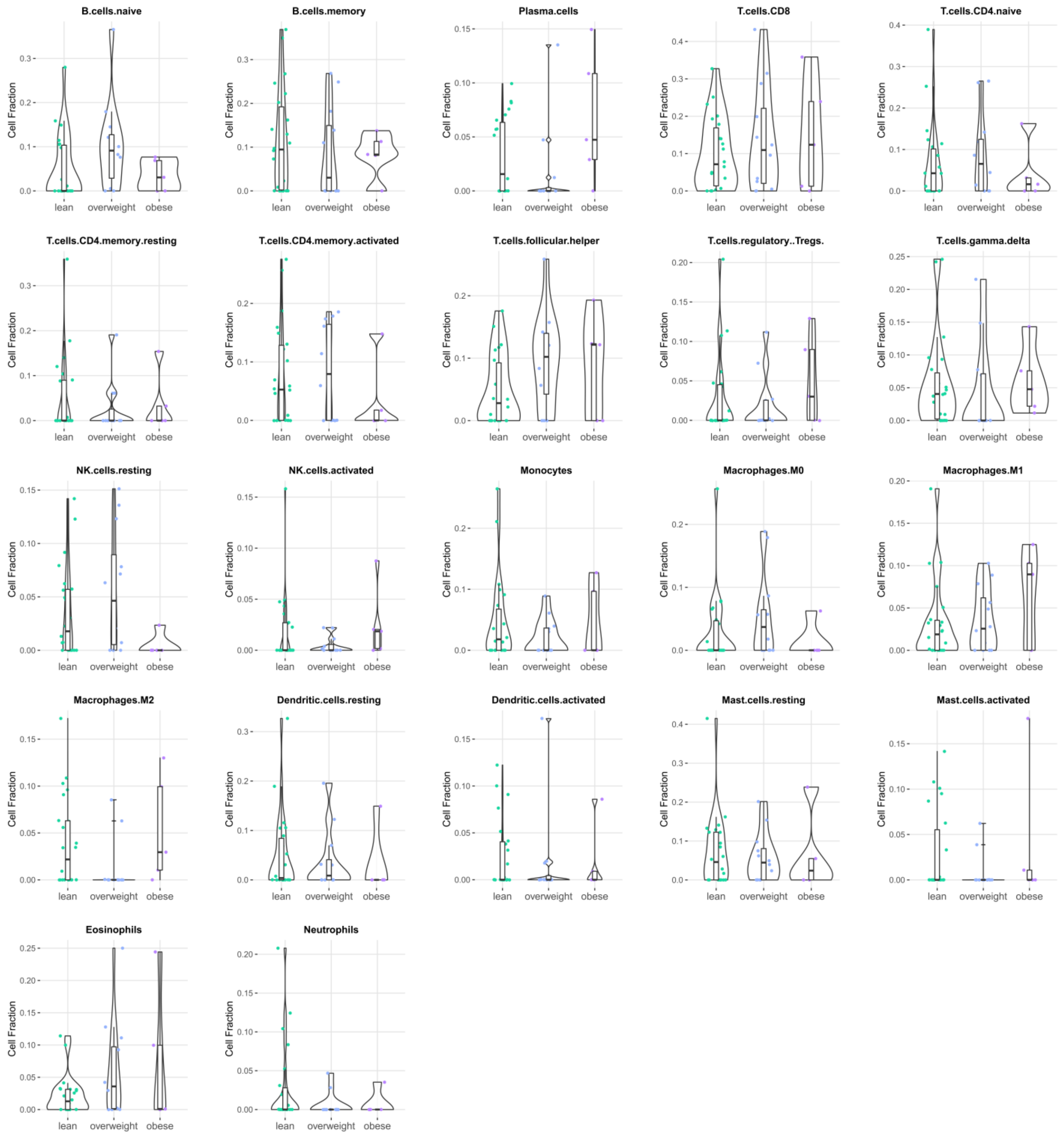

Supplementary Figure 21. Cell fractions of 22 immune cell types in the tumor bulk of ILC ER+/HER2- tumors from the MINDACT cohort. Violin plots showing relative frequency of 22 immune cells inferred by deconvolution of the bulk profiling data using CIBERSORTx according to BMI categories (lean, n = 65; overweight, n = 32; obese, n = 7). In each boxplot, the box denotes the range from the 25th to the 75th percentile, the center line indicates the median value, and the whiskers specify the maxima and minima excluding outliers.

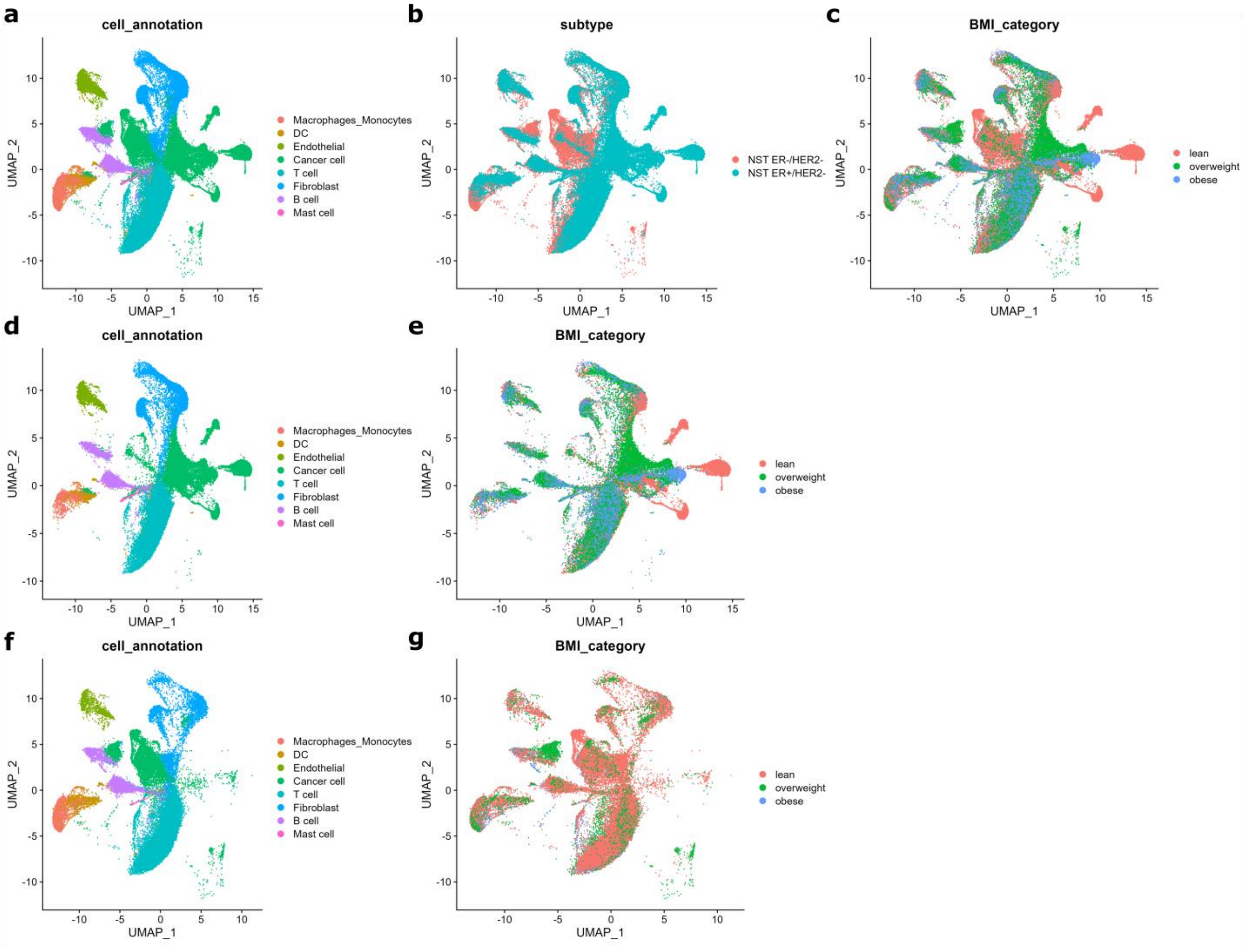

Supplementary Figure 22. Uniform Manifold Approximation and Projection (UMAP) of cells in tumors from NST ER+/HER2- and NST ER-/HER2- patients from the Biokey cohort. a-c UMAP of cells from all patients color-coded according to cell type (a), tumor subtype (b), and BMI category of the patient (c). **d-g** UMAPs of cells from NST ER+/HER2- (d-e) and NST ER-/HER2- (f-g) color-coded according to cell type and BMI category.

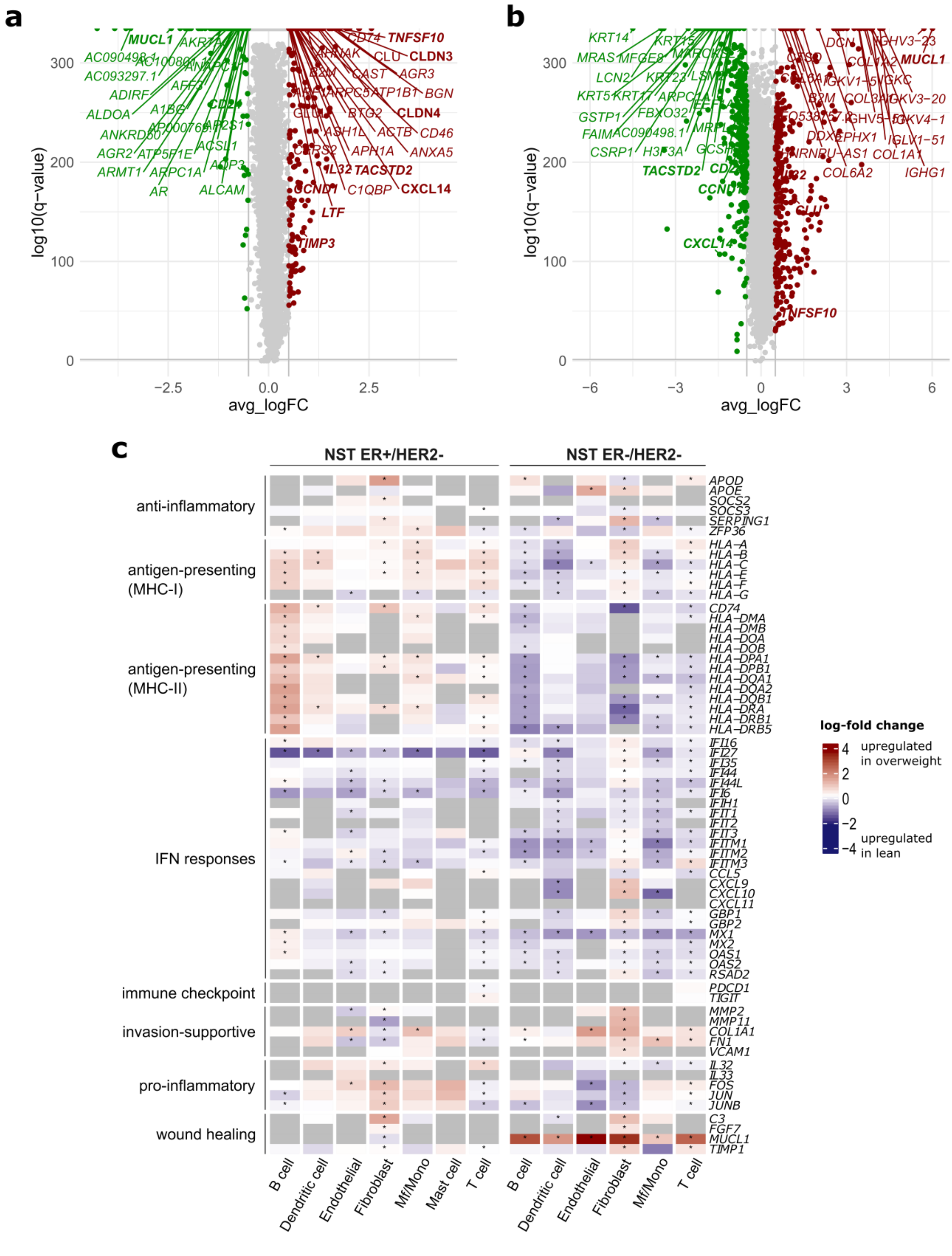

Supplementary Figure 23. Cell type-specific differential gene expression in overweight versus lean patients with NST ER+/HER2- and NST ER-/HER2- from the BioKey cohort. a-b Volcano plots highlighting cancer cell-specific DEGs between overweight and lean in the NST ER+/HER2- (a) and NST ER-/HER2- (b) subgroups. Genes with absolute logFC > 0.5 and q-value < 0.05 are colored (red: upregulated in cancer cells from overweight patients, green: upregulated in

cancer cells from lean patients). The top 20 up-regulated (sorted by q-value), 20 down-regulated genes, and genes discussed in the main text (in bold) are labeled. **c** Heatmaps showing differential expression of a selection of genes involved in several immune and cancer pathways in non-malignant cell populations. The cell color is scaled based on the log-fold change (logFC) values (overweight vs lean) estimated by the MAST test. Gray cells indicate genes not being tested due to expression in less than 10% of the corresponding cell type in both BMI categories. *, q-value < 0.05

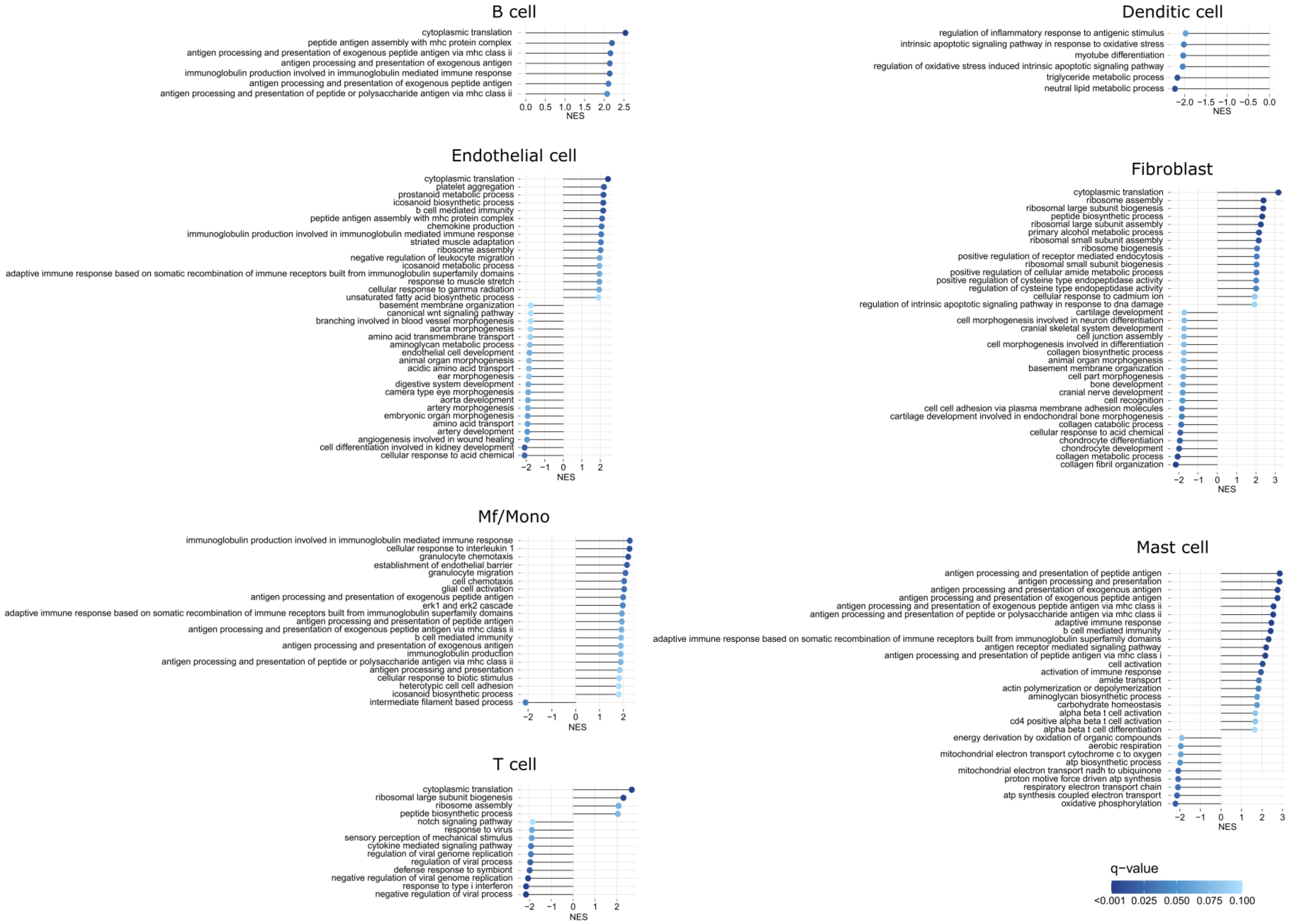
B cell
cytoplasmic translation
peptide antigen assembly with mhc protein complex
antigen processing and presentation of exogenous peptide antigen via mhc class ii
antigen processing and presentation of exogenous antigen
immunoglobulin production involved in immunoglobulin mediated immune response
antigen processing and presentation of exogenous peptide antigen
antigen processing and presentation of peptide or polysaccharide antigen via mhc class ii
0.0 0.5 1.0 1.5 2.0 2.5
NES
Dendritic cell
regulation of inflammatory response to antigenic stimulus
intrinsic apoptotic signaling pathway in response to oxidative stress
myotube differentiation
regulation of oxidative stress induced intrinsic apoptotic signaling pathway
triglyceride metabolic process
neutral lipid metabolic process
−2.0 −1.5 −1.0 −0.5 0.0
NES
Endothelial cell
cytoplasmic translation
platelet aggregation
prostanoid metabolic process
icosanoid biosynthetic process
b cell mediated immunity
peptide antigen assembly with mhc protein complex
chemokine production
immunoglobulin production involved in immunoglobulin mediated immune response
striated muscle adaptation
ribosome assembly
negative regulation of leukocyte migration
icosanoid metabolic process
adaptive immune response based on somatic recombination of immune receptors built from immunoglobulin superfamily domains
response to muscle stretch
cellular response to gamma radiation
unsaturated fatty acid biosynthetic process
basement membrane organization
canonical wnt signaling pathway
branching involved in blood vessel morphogenesis
aorta morphogenesis
amino acid transmembrane transport
aminoglycan metabolic process
endothelial cell development
animal organ morphogenesis
acidic amino acid transport
ear morphogenesis
digestive system development
camera type eye morphogenesis
aorta development
artery morphogenesis
embryonic organ morphogenesis
amino acid transport
artery development
angiogenesis involved in wound healing
cell differentiation involved in kidney development
cellular response to acid chemical
−2 −1 0 1 2
NES
Fibroblast
cytoplasmic translation
ribosome assembly
ribosomal large subunit biogenesis
peptide biosynthetic process
ribosomal large subunit assembly
primary alcohol metabolic process
ribosomal small subunit assembly
ribosome biogenesis
positive regulation of receptor mediated endocytosis
ribosomal small subunit biogenesis
positive regulation of cellular amide metabolic process
positive regulation of cysteine type endopeptidase activity
regulation of cysteine type endopeptidase activity
cellular response to cadmium ion
regulation of intrinsic apoptotic signaling pathway in response to dna damage
cartilage development
cell morphogenesis involved in neuron differentiation
cranial skeletal system development
cell junction assembly
cell morphogenesis involved in differentiation
collagen biosynthetic process
animal organ morphogenesis
basement membrane organization
cell part morphogenesis
bone development
cranial nerve development
cell recognition
cell cell adhesion via plasma membrane adhesion molecules
cartilage development involved in endochondral bone morphogenesis
collagen catabolic process
cellular response to acid chemical
chondrocyte differentiation
chondrocyte development
collagen metabolic process
collagen fibril organization
−2 −1 0 1 2 3
NES
Mf/Mono
immunoglobulin production involved in immunoglobulin mediated immune response
cellular response to interleukin 1
granulocyte chemotaxis
establishment of endothelial barrier
granulocyte migration
cell chemotaxis
glial cell activation
antigen processing and presentation of exogenous peptide antigen
erk1 and erk2 cascade
adaptive immune response based on somatic recombination of immune receptors built from immunoglobulin superfamily domains
antigen processing and presentation of peptide antigen
antigen processing and presentation of exogenous peptide antigen via mhc class ii
b cell mediated immunity
antigen processing and presentation of exogenous antigen
immunoglobulin production
antigen processing and presentation of peptide or polysaccharide antigen via mhc class ii
antigen processing and presentation
cellular response to biotic stimulus
heterotypic cell cell adhesion
icosanoid biosynthetic process
intermediate filament based process
−2 −1 0 1 2
NES
Mast cell
antigen processing and presentation of peptide antigen
antigen processing and presentation
antigen processing and presentation of exogenous antigen
antigen processing and presentation of exogenous peptide antigen
antigen processing and presentation of exogenous peptide antigen via mhc class ii
antigen processing and presentation of peptide or polysaccharide antigen via mhc class ii
adaptive immune response
b cell mediated immunity
adaptive immune response based on somatic recombination of immune receptors built from immunoglobulin superfamily domains
antigen receptor mediated signaling pathway
antigen processing and presentation of peptide antigen via mhc class i
cell activation
activation of immune response
amide transport
actin polymerization or depolymerization
aminoglycan biosynthetic process
carbohydrate homeostasis
alpha beta t cell activation
cd4 positive alpha beta t cell activation
alpha beta t cell differentiation
energy derivation by oxidation of organic compounds
aerobic respiration
mitochondrial electron transport cytochrome c to oxygen
atp biosynthetic process
mitochondrial electron transport nadh to ubiquinone
proton motive force driven atp synthesis
respiratory electron transport chain
atp synthesis coupled electron transport
oxidative phosphorylation
−2 −1 0 1 2 3
NES
T cell
cytoplasmic translation
ribosomal large subunit biogenesis
ribosome assembly
peptide biosynthetic process
notch signaling pathway
response to virus
sensory perception of mechanical stimulus
cytokine mediated signaling pathway
regulation of viral genome replication
regulation of viral process
defense response to symbiont
negative regulation of viral genome replication
response to type i interferon
negative regulation of viral process
−2 −1 0 1 2
NES
q−value
<0.001 0.025 0.050 0.075 0.100

Supplementary Figure 24. Gene set enrichment analysis (GSEA) on Gene Ontology biological process (GOBP) gene sets of non-malignant cells from obese versus lean patients with NST ER+/HER2- from the BioKey cohort. Lollipop plots displaying top 20 upregulated (sorted by q-value, NES > 0) and top 20 downregulated (NES < 0) GOBP gene sets according to BMI category (obese vs lean) detected by GSEA (q-value < 0.1). p-values were computed by permutation and adjusted for multiple testing using the Benjamin-Hochberg method (presented as q-values).

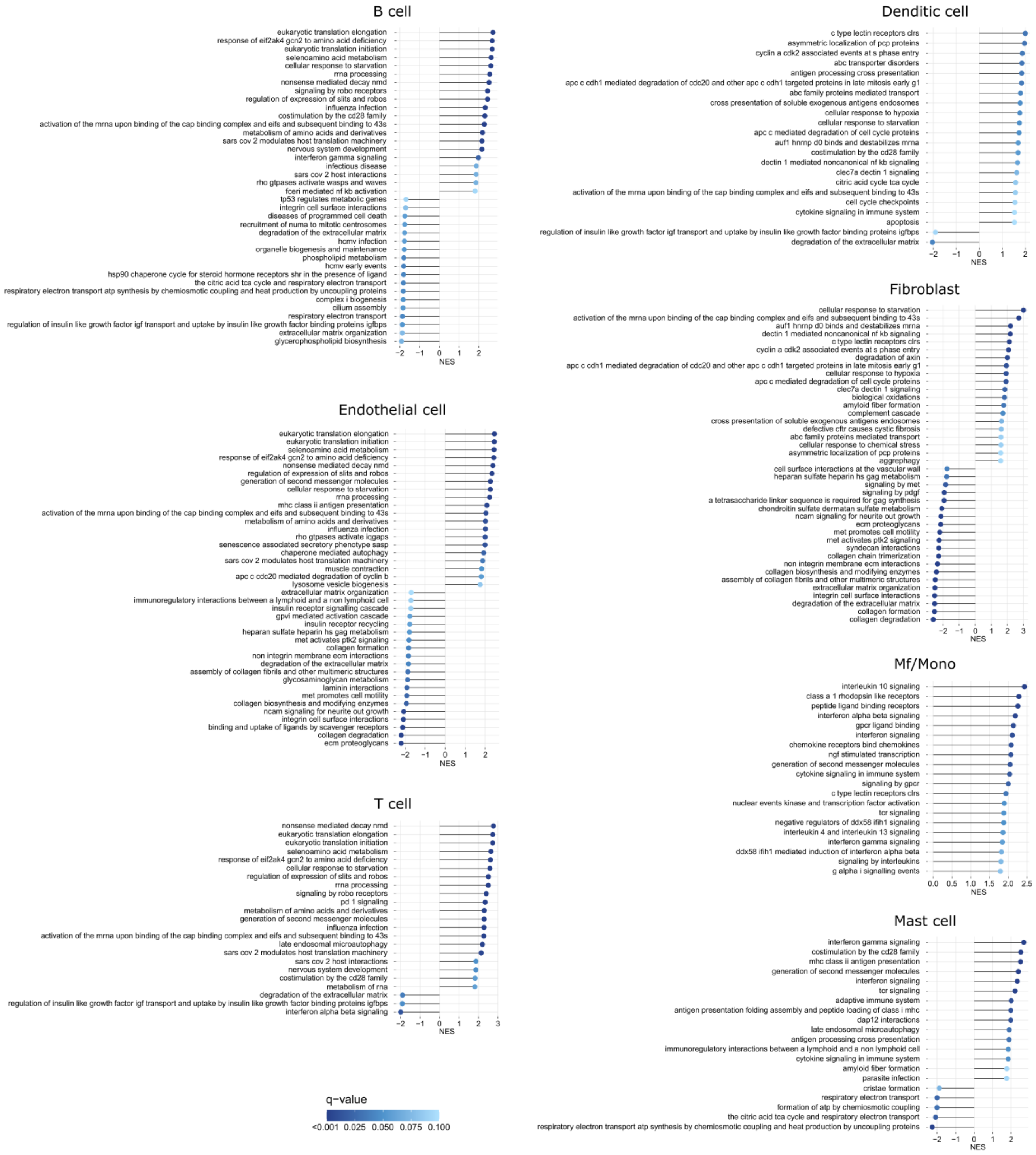

Supplementary Figure 25. Gene set enrichment analysis (GSEA) on REACTOME gene sets of non-malignant cells from obese versus lean patients with NST ER+/HER2- from the BioKey cohort. Lollipop plots displaying top 20 upregulated (sorted by q-value, NES > 0) and top 20 downregulated (NES < 0) REACTOME gene sets according to BMI category (obese vs lean) detected by GSEA (q-value < 0.1). p-values

were computed by permutation and adjusted for multiple testing using the Benjamin-Hochberg method (presented as q-values).

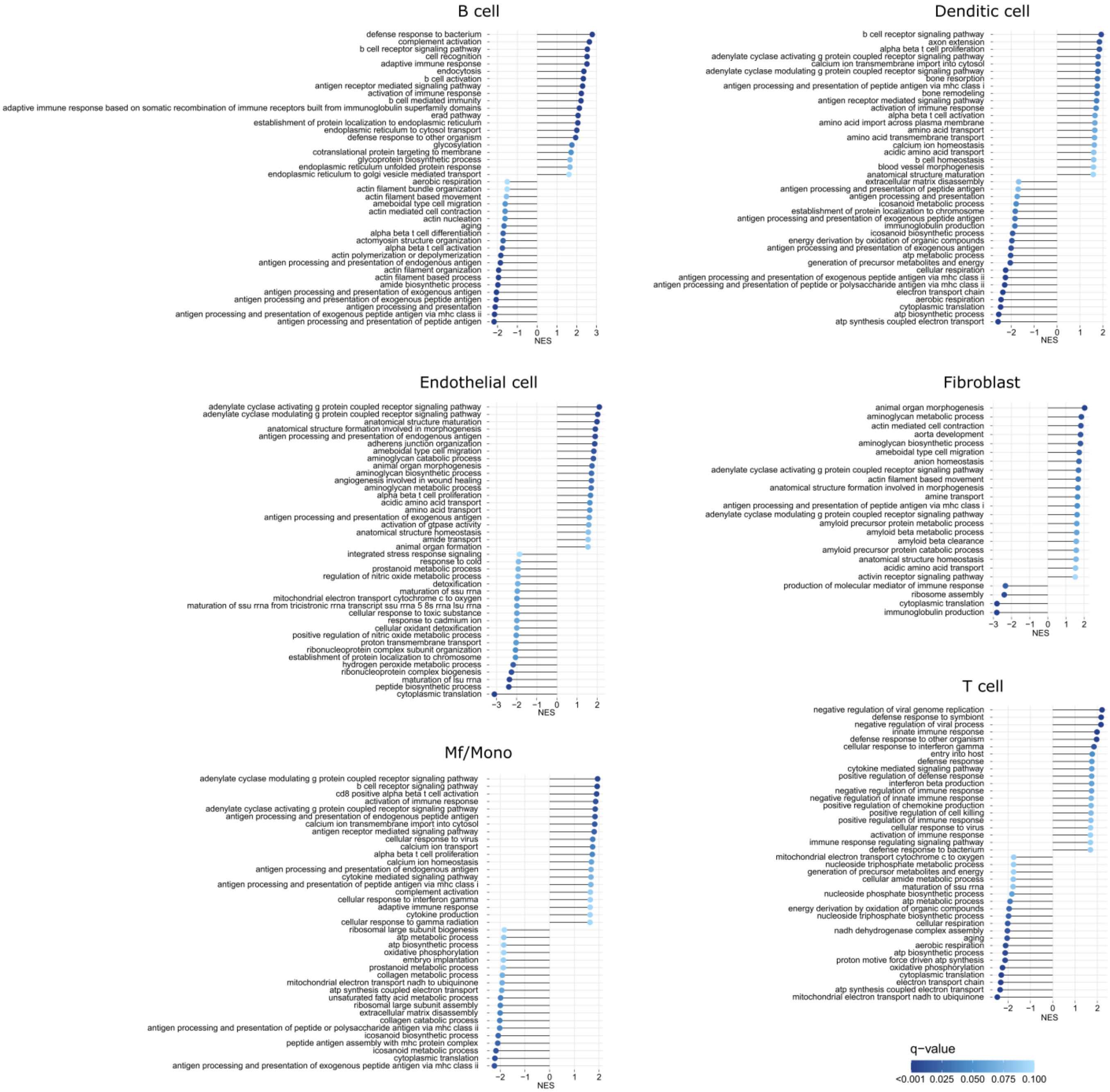

Supplementary Figure 26. Gene set enrichment analysis (GSEA) on GO biological process gene sets of non-malignant cells from obese versus lean patients with NST ER-/HER2- from the BioKey cohort. Lollipop plots displaying top 20 upregulated (sorted by q-value, NES > 0) and top 20 downregulated (NES < 0) GOBP gene sets according to BMI category (obese vs lean) detected by GSEA (q-value < 0.1). p-values were computed by permutation and adjusted for multiple testing using the Benjamin-Hochberg method (presented as q-values).

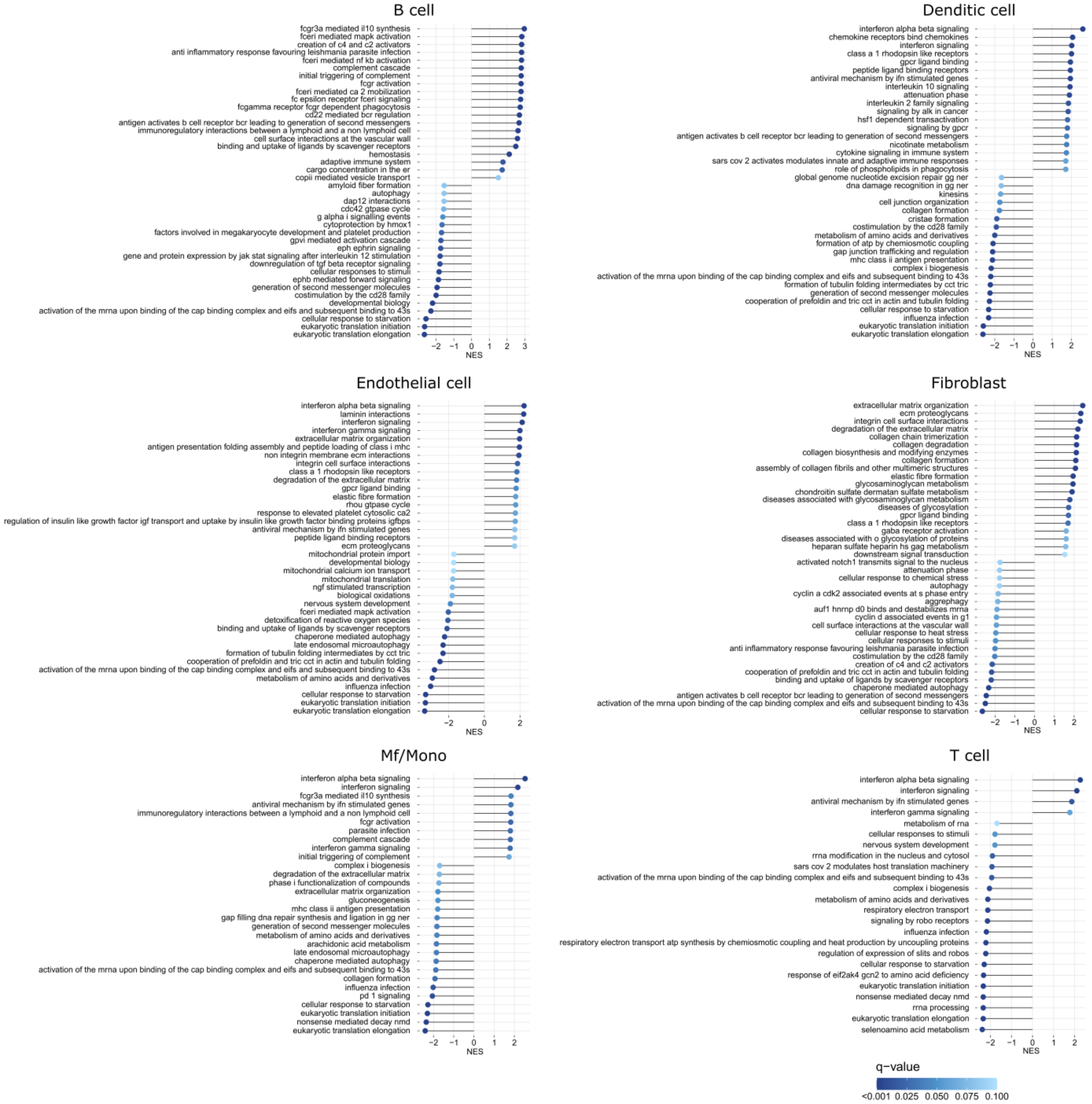

Supplementary Figure 27. Gene set enrichment analysis (GSEA) on REACTOME gene sets of non-malignant cells from obese versus lean patients with NST ER-/HER2- from the BioKey cohort. Lollipop plots displaying top 20 upregulated (sorted by q-value, NES > 0) and top 20 downregulated (NES < 0) REACTOME gene sets according to BMI category (obese vs lean) detected by GSEA (q-value < 0.1). p-values were computed by permutation and adjusted for multiple testing using the Benjamin-Hochberg method (presented as q-values).

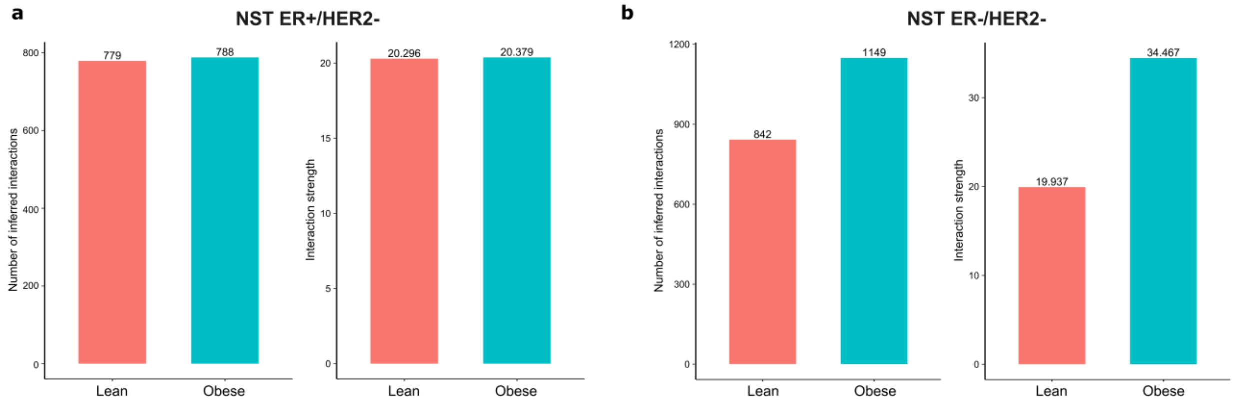

Supplementary Figure 28. Total intercellular interactions in tumors from lean and obese patients in the BioKey cohort. a-b Sum of signaling interactions in terms of number and strength inferred by CellChat in NST ER+/HER2- (a) and NST ER-/HER2- (b) tumors from lean and obese patients.

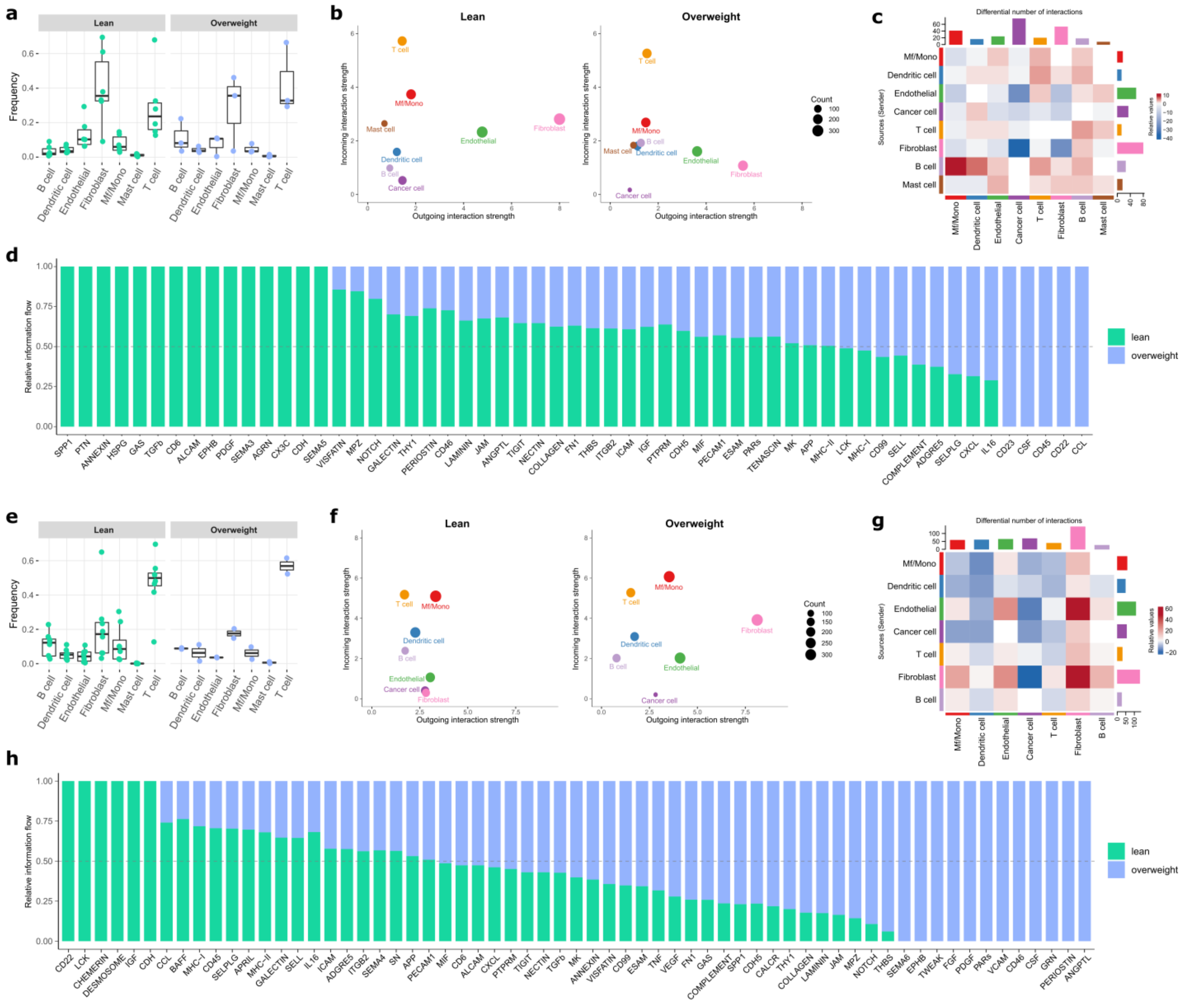

Supplementary Figure 29. Differences in the cell composition and intercellular interactions within the tumor microenvironment between overweight and lean patients with NST ER+/HER2- (a-d) and NST ER-/HER2- (e-h) tumors from the BioKey cohort. a, e Frequency of non-malignant cell types relative to the non-malignant cell pool for lean and overweight patients (NST ER+/HER2- lean, n = 6; overweight, n = 3; NST ER-/HER2- lean, n = 8; overweight, n = 2). In each boxplot, the box denotes the range from the 25th to the 75th percentile, the center line indicates the median value, and the whiskers specify the maxima and minima excluding outliers. **b, f** CellChat-derived outgoing and incoming interaction strength from and to cancer cells and non-malignant cells in tumors from lean and overweight patients. **c, g** Differential number of interactions detected by CellChat between lean and overweight. **d, h** Relative Information flow of signaling pathways in the intercellular communication network in tumors from lean and overweight patients. The relative information flow was estimated by the sum of CellChat-derived communication

probability between all pairs of cell compartments in the network. Signaling pathways with non-zero information in at least one of the BMI categories are shown.
