# Supplementary material for: Obesity-associated changes in molecular biology of primary breast cancer
Source: Nat Commun. 2023 Jul 21;14:4418. doi: 10.1038/s41467-023-39996-z (PMC10361985; doi:10.1038/s41467-023-39996-z)
Supplement: Supplementary file 2 — Description of Additional Supplementary Files [file 41467_2023_39996_MOESM2_ESM.pdf]

Description of Additional Supplementary Files

File Name: Supplementary Data 1
Description: **Clinical and pathological characteristics of patients with available non-underweight BMI (investigated subset) and all patients in cohorts with incomplete BMI data.** Association between clinicopathological variables and BMI availability was assessed using Fisher's exact test for each cohort. p-values were not adjusted for multiple testing. All statistical tests were two-sided

File Name: Supplementary Data 2
Description: **Clinical and pathological characteristics of patients with available non-underweight BMI in all cohorts.** Association between clinicopathological variables and cohort was assessed using Fisher's exact test for cohorts that were not histology specific (i.e. METABRIC, ICGC, and MINDACT), and two cohorts that were merged in subsequent analyses of genomic alteration data (i.e. METABRIC and ICGC). p-values were not adjusted for multiple testing. All statistical tests were two-sided. The Biokey cohort was excluded from the statistical test due to a small size.

File Name: Supplementary Data 3
Description: **Clinical and pathological characteristics according to BMI category of patients in all cohorts.** Association between clinicopathological variables and categorical BMI was assessed using Fisher's exact test. p-values were not adjusted for multiple testing. All statistical tests were two-sided. The Biokey cohort was excluded from the statistical test due to a small size.

File Name: Supplementary Data 4
Description: **List of driver genes used to investigate the association of genomic alterations with BMI.** Genes harboring base substitutions and indels, or CNAs reported as BC driver events by Nik-Zainal et al.

File Name: Supplementary Data 5
Description: **Oncogenic base substitutions and indels in tumors from METABRIC and ICGC.**

File Name: Supplementary Data 6
Description: **Gene-level copy number alterations in tumors from METABRIC and ICGC.**

File Name: Supplementary Data 7
Description: **Association of recurrent gene mutations with continuous BMI.** Merged data from two cohorts, METABRIC and ICGC, was used for analyses of the NST ER+/HER2- and NST ER-/HER2- subtypes, and from the ELBC cohort for the ILC ER+/HER2- subtype. Gene mutations with at least 5 events detected in the respective sub cohorts were evaluated. Linear association was assessed using Firth's logistic regression models: Model 1 was adjusted for cohort; Model 2 was adjusted for cohort, age, and tumor grade. Potential non-linear association was explored using generalized additive models (GAM) whereby models with (SPLINE) and without (noSPLINE) a spline term were compared using two metrics, AIC and a LR test. All

statistical tests were two-sided.

File Name: Supplementary Data 8
Description: **Association of prevalence of hotspot substitutions and indels with categorical BMI.** Merged data from two cohorts, METABRIC and ICGC, was used for analyses of the NST ER+/HER2- and NST ER-/HER2-subtypes. Substitutions and indels with at least one event detected in tumors from lean patients, and at least one event detected in tumors from either overweight or obese patients, in the respective sub-cohorts, were tested. p-values were derived from Fisher's exact tests. All statistical tests were two-sided.

File Name: Supplementary Data 9
Description: **Association of recurrent gene-level CNAs with continuous BMI.** Merged data from two cohorts, METABRIC and ICGC, was used for analyses of the NST ER+/HER2- and NST ER-/HER2- subtypes, and from the ELBC cohort for the ILC ER+/HER2-subtype. Gene-level CNAs with at least 10 events detected in the respective cohorts were evaluated. Linear association was assessed using Firth's logistic regression models: Model 1 was adjusted for cohort; Model 2 was adjusted for cohort, age, and tumor grade. Potential non-linear association was explored using generalized additive models (GAM) whereby models with (SPLINE) and without (noSPLINE) a spline term were compared using two metrics, AIC and a LR test. All statistical tests were two-sided.

File Name: Supplementary Data 10
Description: **Association of recurrent gene-level CNAs with categorical BMI.** Merged data from two cohorts, METABRIC and ICGC, was used for analyses of the NST ER+/HER2- and NST ER-/HER2- subtypes, and from the ELBC cohort for the ILC ER+/HER2- subtype. Association was assessed using Firth's logistic regression models: Model 1 was adjusted for cohort; Model 2 was adjusted for cohort, age, and tumor grade. Gene-level CNAs with at least 10 events detected in the respective cohorts were evaluated. All statistical tests were two-sided.

File Name: Supplementary Data 11
Description: **Association of genome instability and mutational and rearrangement signatures with continuous and categorical BMI.** Corresponding data from the ICGC cohort were used for analyses of the NST ER+/HER2- and NST ER-/HER2- subtypes. Association was assessed using linear regression models: Model 1 was univariable; Model 2 was adjusted for age, and tumor grade. All statistical tests were two-sided.

File Name: Supplementary Data 12
Description: **Cell type-specific differentially expressed genes in NST ER+/HER2- and NST ER-/HER2- tumors from obese patients versus lean patients.** Data from the BioKey cohort was used for analyses. Mast cells were not evaluated for the NST ER-/HER2- subtype due to low absolute cell counts. Genes with absolute logFC > 0.5 and q value < 0.05 are shown. A logFC greater than 0 indicates an overexpression of the respective gene in the respective cell type in tumors from obese patients, and vice versa. p-values were determined for logFC estimated by the MAST test and adjusted for multiple testing (presented as q-values). All statistical tests were two-sided.

File Name: Supplementary Data 13

Description: **Cell type-specific differentially expressed genes in NST ER+/HER2- and NST ER-/HER2- tumors from overweight patients versus lean patients.** Data from the BioKey cohort was used for analyses. Mast cells were not evaluated for the NST ER-/HER2- subtype due to low absolute cell counts. Genes with absolute logFC > 0.5 and q-value < 0.05 are shown. A logFC greater than 0 indicates an overexpression of the respective gene in the respective cell type in tumors from overweight patients, and vice versa. p-values were determined for logFC estimated by the MAST test and adjusted for multiple testing (presented as q-values). All statistical tests were two-sided.

File Name: Supplementary Data 14

Description: **Cell type-specific differentially enriched GOBP gene sets in NST ER+/HER2- and NST ER- /HER2- tumors from obese patients versus lean patients.** Data from the BioKey cohort was used for analyses. Mast cells were not evaluated for the NST ER-/HER2- subtype due to low absolute cell counts. Gene sets with q-value < 0.1 are shown. A NES greater than 0 indicates an enrichment of the respective gene set in the respective cell type in tumors from obese patients, and vice versa. p-values were computed by permutation and adjusted for multiple testing (presented as q-values). All statistical tests were two-sided.

File Name: Supplementary Data 15

Description: **Cell type-specific differentially enriched REACTOME gene sets in NST ER+/HER2- and NST ER-/HER2- tumors from obese patients versus lean patients.** Data from the BioKey cohort was used for analyses. Mast cells were not evaluated for the NST ER-/HER2- subtype due to low absolute cell counts. Gene sets with q-value < 0.1 are shown. A NES greater than 0 indicates an enrichment of the respective gene set in the respective cell type in tumors from obese patients, and vice versa. p-values were computed by permutation and adjusted for multiple testing (presented as q-values).

File Name: Supplementary Data 16

Description: **Cell type-specific differentially enriched GOBP gene sets in NST ER+/HER2- and NST ER- /HER2- tumors from overweight patients versus lean patients.** Data from the BioKey cohort was used for analyses. Mast cells were not evaluated for the NST ER-/HER2- subtype due to low absolute cell counts. Gene sets with q-value < 0.1 are shown. A NES greater than 0 indicates an enrichment of the respective gene set in the respective cell type in tumors from overweight patients, and vice versa. p-values were computed by permutation and adjusted for multiple testing (presented as q-values).

File Name: Supplementary Data 17

Description: **Cell type-specific differentially enriched REACTOME gene sets in NST ER+/HER2- and NST ER-/HER2-tumors from overweight patients versus lean patients.** Data from the BioKey cohort was used for analyses. Mast cells were not evaluated for the NST ER-/HER2- subtype due to low absolute cell counts. Gene sets with q-value < 0.1 are shown. A NES greater than 0 indicates an enrichment of the respective gene set in the respective cell type in tumors from overweight patients, and vice versa. p-values were computed by permutation and adjusted for multiple testing (presented as q-values)

File Name: Supplementary Data 18
Description: **Cell counts in investigated cellular compartments in NST ER+/HER2- and NST ER-/HER2- tumors according to BMI category in the Biokey cohort**

File Name: Supplementary Data 19
Description: **Absolute counts and relative frequencies of investigated cellular compartments in individual NST ER+/HER2- and NST ER-/HER2- tumors in the Biokey cohort.** Frequency of each investigated cell types was computed relative to either the total cell pool or total non-malignant cell pool for each tumor.
