# Supplementary material for: Obesity-associated changes in molecular biology of primary breast cancer
Source: Nat Commun. 2023 Jul 21;14:4418. doi: 10.1038/s41467-023-39996-z (PMC10361985; doi:10.1038/s41467-023-39996-z)
Supplement: Supplementary file 3 — Reporting Summary [file 41467_2023_39996_MOESM3_ESM.pdf]

nature portfolio

Corresponding author(s): Christine Desmedt
Last updated by author(s): May 26, 2023

Reporting Summary

Nature Portfolio wishes to improve the reproducibility of the work that we publish. This form provides structure for consistency and transparency in reporting. For further information on Nature Portfolio policies, see our Editorial Policies and the Editorial Policy Checklist.

Statistics

For all statistical analyses, confirm that the following items are present in the figure legend, table legend, main text, or Methods section.

n/a	Confirmed	
☐	☒	The exact sample size (*n*) for each experimental group/condition, given as a discrete number and unit of measurement
☐	☒	A statement on whether measurements were taken from distinct samples or whether the same sample was measured repeatedly
☐	☒	The statistical test(s) used AND whether they are one- or two-sided<br>*Only common tests should be described solely by name; describe more complex techniques in the Methods section.*
☐	☒	A description of all covariates tested
☐	☒	A description of any assumptions or corrections, such as tests of normality and adjustment for multiple comparisons
☐	☒	A full description of the statistical parameters including central tendency (e.g. means) or other basic estimates (e.g. regression coefficient) AND variation (e.g. standard deviation) or associated estimates of uncertainty (e.g. confidence intervals)
☐	☒	For null hypothesis testing, the test statistic (e.g. *F*, *t*, *r*) with confidence intervals, effect sizes, degrees of freedom and *P* value noted<br>*Give P values as exact values whenever suitable.*
☒	☐	For Bayesian analysis, information on the choice of priors and Markov chain Monte Carlo settings
☒	☐	For hierarchical and complex designs, identification of the appropriate level for tests and full reporting of outcomes
☒	☐	Estimates of effect sizes (e.g. Cohen's *d*, Pearson's *r*), indicating how they were calculated

Our web collection on statistics for biologists contains articles on many of the points above.

Software and code

Policy information about availability of computer code

Data collection	Data were retrieved from publications and open-access data portals, or through data sharing agreement, hence no specific software was needed for data collection.
Data analysis	Analyses of somatic genomic data were performed using R (4.1.1) with the following packages: logistf (v1.24.1), Rediscover (v0.3.0).<br>Analyses of bulk gene expression data were performed using R (v4.1.1) with the following packages: limma (v3.48.3), GSVA (v1.40.1).<br>Analyses of single-cell RNA-seq data were performed using R (v4.1.1) with the following packages: Seurat (v4.1.1), CellChat (v1.5.0).<br>Stand-alone softwares: GSEA (v4.1.0, for Gene Set Enrichment Analysis).<br>Data visualization was performed using R (v4.1.1) with the following packages: ggplot2 (v3.4.0), forestplot (v2.0.1), ComplexHeatmap (v2.8.0), circlize (v0.4.15), factoextra (v1.0.7), ggbiplot (v0.55), ggrepel (v0.9.1), gridExtra (v2.3)

For manuscripts utilizing custom algorithms or software that are central to the research but not yet described in published literature, software must be made available to editors and reviewers. We strongly encourage code deposition in a community repository (e.g. GitHub). See the Nature Portfolio guidelines for submitting code & software for further information.

Data

Policy information about availability of data

All manuscripts must include a data availability statement. This statement should provide the following information, where applicable:

- Accession codes, unique identifiers, or web links for publicly available datasets
- A description of any restrictions on data availability
- For clinical datasets or third party data, please ensure that the statement adheres to our policy

Data from the ICGC cohort (project BRCA-EU) can be accessed through the ICGC Data Portal [https://dcc.icgc.org/projects/BRCA-EU] and through published data (Nik-Zainal et al. Nature 2016). Data from METABRIC can be accessed through cBioPortal [https://www.cbioportal.org/study/summary?id=brca_metabric] and through published data (Curtis et al. Nature 2012, Mukherjee et al. NPJ Breast Cancer 2018). Data from ELBC can be accessed through published data (Desmedt et al. JCO 2016) and Gene Expression Omnibus (accession number GSE88770 [https://www.ncbi.nlm.nih.gov/geo/query/acc.cgi?acc=GSE88770]). BMI data for the ICGC, METABRIC, and ELBC cohorts were additionally collected and are accessible via the CodeOcean capsule (see Code availability). Data from MINDACT can be accessed through the EORTC ([https://www.eortc.org/data-sharing/]). Download of the read count data per individual patient from BioKey is publicly available at http://biokey.lambrechtslab.org. Raw sequencing reads of the scRNA-seq experiments have been deposited in the European Genome-phenome Archive (EGA) under study no. EGAS00001004809 [https://ega-archive.org/studies/EGAS00001004809] (with a summary of the BioKey study and patient characteristics) and with data accession no. EGAD00001006608 [https://ega-archive.org/datasets/EGAD00001006608] (to access the data itself under restricted access). Requests for accessing raw sequencing reads and processed data will be reviewed by the UZLeuven-VIB data access committee. Any data shared will be released via a Data Transfer Agreement that will include the necessary conditions to guarantee protection of personal data (according to European GDPR law).

Human research participants

Policy information about studies involving human research participants and Sex and Gender in Research.

Reporting on sex and gender	Information on sex was not included in the published data for the METABRIC cohort. 259 female and 2 male patients from the ICGC cohort were included in the analyses. The ELBC, MINDACT, and Biokey cohorts were strictly all-female cohorts. Given the negligible known number of male patients in the study cohorts, our findings apply to breast cancer in female.
Population characteristics	This study analyzed data from patients with early breast cancer having non-underweight BMI. Samples were collected from primary breast tumors prior to any treatment. Baseline characteristics of all patients included in the analyses are provided in Supplementary Table 2.
Recruitment	This study retrospectively analyzed data from existing studies. No recruitment was done within the scope of this study.
Ethics oversight	Ethical approval was granted for each of the source studies. Data were acquired for the purpose of this study through publications and open-access data portals for the ICGC, METABRIC, ELBC, and Biokey cohorts. Ethical compliance for the use of data from the MINDACT cohort was ensured through a Data Transfer Agreement approved by the EORTC.

Note that full information on the approval of the study protocol must also be provided in the manuscript.

Field-specific reporting

Please select the one below that is the best fit for your research. If you are not sure, read the appropriate sections before making your selection.

☒ Life sciences ☐ Behavioural & social sciences ☐ Ecological, evolutionary & environmental sciences

For a reference copy of the document with all sections, see nature.com/documents/nr-reporting-summary-flat.pdf

Life sciences study design

All studies must disclose on these points even when the disclosure is negative.

Sample size	There was no sample size computation performed in the scope of this study provided its retrospective setup and exploratory nature. Data of all subjects eligible for analyses were analyzed.
Data exclusions	The following patients were excluded from the analyses: Patients with unknown BMI; Patients with BMI < 18.5kg/m2 (underweight); Patients with tumors histologically defined as any category other than invasive breast carcinoma of no special type or invasive lobular carcinoma; Patients with unknown estrogen receptor and HER2 status; Patients with HER2-positive tumors.
Replication	This study involves data from multiple retrospective series of breast cancer patients. Each subject (patient) is considered a biological replicate. The statistical methods used took into consideration the variation between subjects for determination of significance.
Randomization	Randomization is not relevant for this study as it is a retrospective study. Patients were stratified into subgroups based on several baseline characteristics of their tumors: histology, estrogen receptor status, and HER2 status.
Blinding	Blinding is not relevant for this study as it is a retrospective study, without any allocation of intervention.

Reporting for specific materials, systems and methods

We require information from authors about some types of materials, experimental systems and methods used in many studies. Here, indicate whether each material, system or method listed is relevant to your study. If you are not sure if a list item applies to your research, read the appropriate section before selecting a response.

Materials & experimental systems

n/a	Involved in the study
☒	☐ Antibodies
☒	☐ Eukaryotic cell lines
☒	☐ Palaeontology and archaeology
☒	☐ Animals and other organisms
☒	☐ Clinical data
☒	☐ Dual use research of concern

Methods

n/a	Involved in the study
☒	☐ ChIP-seq
☒	☐ Flow cytometry
☒	☐ MRI-based neuroimaging
